# Epitope Mapping and Binding Assessment by Solid-State NMR Provide a Way for the Development of Biologics under the Quality by Design Paradigm

**DOI:** 10.1021/jacs.2c03232

**Published:** 2022-05-26

**Authors:** Domenico Rizzo, Linda Cerofolini, Stefano Giuntini, Luisa Iozzino, Carlo Pergola, Francesca Sacco, Angelo Palmese, Enrico Ravera, Claudio Luchinat, Fabio Baroni, Marco Fragai

**Affiliations:** † Magnetic Resonance Center (CERM), University of Florence, Via L. Sacconi 6, 50019 Sesto Fiorentino, Italy; ‡ Department of Chemistry “Ugo Schiff”, University of Florence, Via della Lastruccia 3, 50019 Sesto Fiorentino, Italy; § Consorzio Interuniversitario Risonanze Magnetiche di Metalloproteine (CIRMMP), Via L. Sacconi 6, 50019 Sesto Fiorentino, Italy; ∥ Analytical Development Biotech Department, Merck Serono S.p.a, Via Luigi Einaudi, 11, 00012 Guidonia, RM, Italy

## Abstract

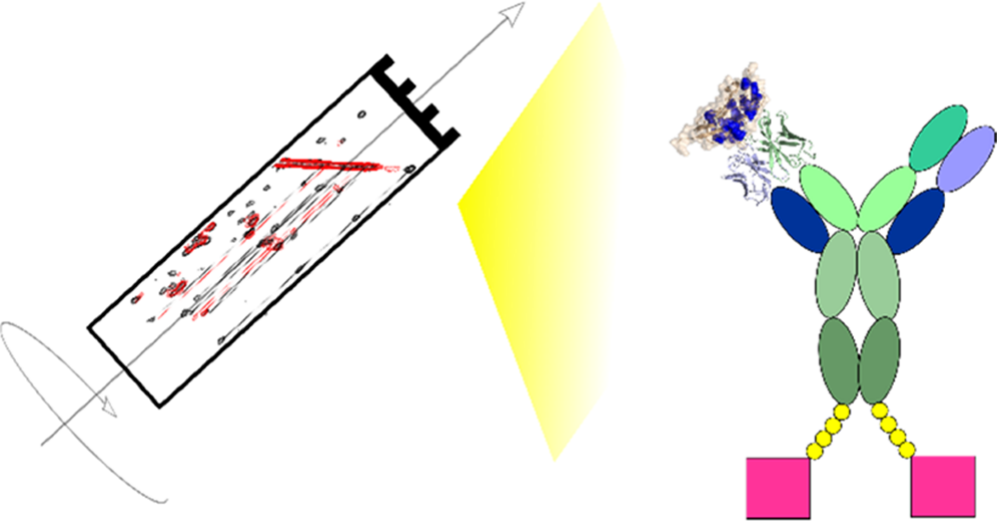

Multispecific biologics are an emerging class of drugs, in which antibodies and/or proteins designed to bind pharmacological targets are covalently linked or expressed as fusion proteins to increase both therapeutic efficacy and safety. Epitope mapping on the target proteins provides key information to improve the affinity and also to monitor the manufacturing process and drug stability. Solid-state NMR has been here used to identify the pattern of the residues of the programmed cell death ligand 1 (PD-L1) ectodomain that are involved in the interaction with a new multispecific biological drug. This is possible because the large size and the intrinsic flexibility of the complexes are not limiting factors for solid-state NMR.

## Introduction

Drug discovery is a long and costly process that has a very low success rate. Structural biology is the game-changer for the identification and optimization of new lead compounds, but the relevance of the structural information that can be gathered is causing structural biology to emerge also for the development of biotherapeutics.^1,2^

As defined by international guidelines, pharmaceutical development should adhere to the Quality by Design paradigm (QbD), described by ICH Q8 (R2)^3^ from the European Medicine Agency (EMA) as a “systematic approach to development that begins with predefined objectives and emphasizes product and process understanding and process control, based on sound science and quality risk management”. This important concept has revolutionized drug development by highlighting the importance of new analytical strategies based on advanced product and process knowledge. Developing a drug under the QbD paradigm not only aims at improving the quality and safety of pharmaceutical products but also at increasing the success rate by improving Critical Quality Attributes risk assessments, leading to more focused control strategies and release testing panels.

Monoclonal antibodies (mAbs) are, to date, the major class of biological drugs approved for the treatment of a large variety of pathologies, and new engineering solutions have solved most of the serious problems encountered in the therapeutic use of these proteins, improving the interactions with the effector cells, leading to less immunogenic molecules and allowing the selection of high-affinity species.^4,5^ Among these drugs, multispecific biologics obtained by fusing full-length antibodies, fragment antigen-binding (FAB), or other proteins together represent the next generation of biotherapeutics.^6−12^ This entire class of drugs can benefit from structural information obtained by investigating their complexes with the targets, for example, to reshape and optimize the interaction site.^13,14^

Structural information at the atomic level about the macromolecular complexes is routinely obtained using X-ray crystallography,^15,16^ much less so by NMR^17,18^ and, more recently, cryo-electron microscopy.^19,20^ However, the large molecular weight and the flexibility of fusion-derived biotherapeutics often prevent the structural characterization of their complexes with the targets. For instance, a large inherent flexibility makes it difficult to obtain crystals of diffraction quality or cryo-EM reconstruction. At the same time, the large molecular weight of these systems hampers a deep structural characterization by NMR in solution, although NMR is successfully used in the higher-order structure (HOS) assessment.^21−29^ Relevant and complementary information can be obtained from hydrogen–deuterium exchange coupled to mass spectrometry (HDX-MS): characterization of interaction surfaces in protein complexes is one of the strengths of this technique, but complex and extensive method optimization is needed, and data interpretation is not straightforward.^30,31^

Thanks to advances in the instrumentation and in sample preparation, solid-state NMR has reached sufficient maturity to start tackling systems of outstanding complexity, such as biological drugs, vaccine formulations, etc. A few years ago, a pioneering work by the group of Lewandowski reported the solid-state NMR characterization of a precipitated macromolecular complex between the first immunoglobulin binding domain of streptococcal protein G (GB1) and a full-length antibody.^32^ GB1 is a 6 kDa protein^33^ that is extensively used as a standard in solid-state NMR,^34^ and is reported to bind strongly to the crystallizable region fragment and weakly to the antigen-binding fragment of human immunoglobulin G. These results and previous studies on noncrystalline systems suggest that also very large macromolecular systems involving fusion-derived biologics can be characterized by solid-state NMR spectroscopy.^35−62^ One of the advantages of the noncrystalline samples, obtained by sedimentation or equivalently by rehydrating freeze-dried proteins,^63^ is the absence of crystalline (ordered) packing.^45^ Indeed, the shift perturbations due to the contacts among the different protein molecules are averaged over several poses with no energetic preferences and the hydration state of the biomolecules is closer to that present in solution.^63,64^ Therefore, a rehydrated freeze-dried material corresponds to an extremely concentrated solution of the protein, which is intrinsically comparable, for the scope of chemical shift mapping, to the diluted sample used for acquiring solution spectra.^65^

The observation of well-resolved spectra on a noncrystalline system of a small protein is not trivial: in our experience, noncrystalline samples of small proteins—including domains or fragments of therapeutic targets—can provide poor-quality solid-state NMR spectra^63^ that have discouraged so far the use of this strategy in the investigation of pharmaceutical relevant systems and in the development of biologics. Local structural inhomogeneity under magic angle spinning (MAS) conditions is among the possible reasons of the unsatisfactory quality of solid-state spectra recorded on noncrystalline samples of some small proteins. In the case of antibodies, however, since they usually bind a target with very high affinity by establishing an extensive network of interactions, a structural stabilization of the interacting protein is expected.

Programmed cell death 1 (PD-1)/programmed cell death ligand 1 (PD-L1) axis is one of the immune checkpoints that under healthy conditions promote self-tolerance and protect the host from autoimmunity.^66^ However, the PD-1/PD-L1 cascade is also used by several cancer cell lines to avoid the immune response by overexpressing the PD-L1 transmembrane protein on the surface.^67,68^ The ectodomain of PD-L1 is therefore the target for several in-use and in-development antibodies employed in the therapy of cancers overexpressing this protein.^69−72^ In this respect, the assignment of the target protein in complex with biotherapeutics provides the way for a structure-based approach to drug development and manufacturing.

**Figure 1:**
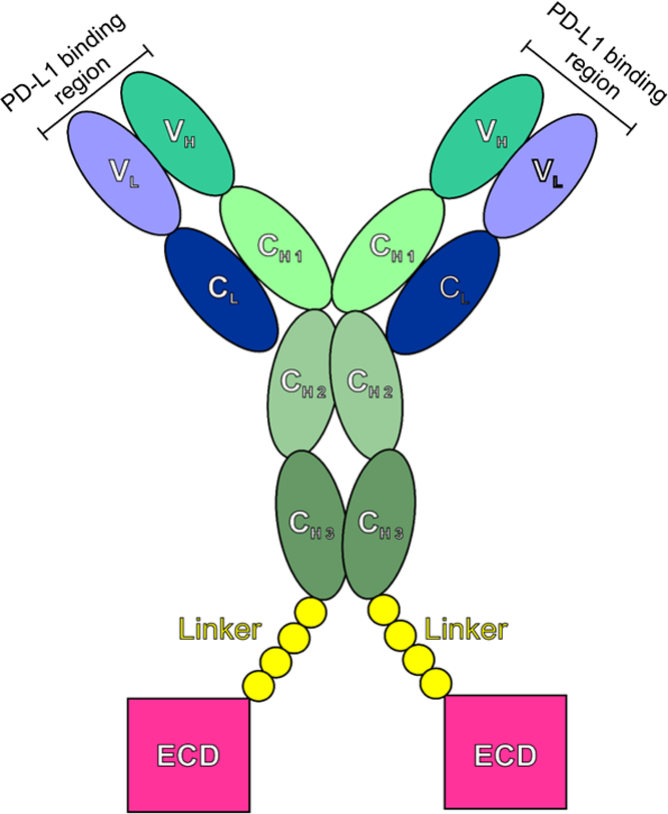
Schematic representation of the anti-PD-L1 fusion protein.

This study explores the interaction between the PD-L1 receptor and an anti-PD-L1 biotherapeutic: an IgG1 fusion protein of about 190 kDa, composed of an extracellular domain (ECD) protein covalently linked via a flexible linker to the C-terminus of each heavy chain of an anti-PD-L1 antibody (Figure 1). Here, we show that the epitope mapping of this Fc-fusion protein on the PD-L1 ectodomain can be achieved by integrating solution and solid-state NMR studies and that the structural information obtained with our approach can be used to provide usable knowledge to develop a biotherapeutic under the Quality by Design paradigm (QbD).

## Methods

### Expression and Purification of [U-^13^C, ^15^N] and [U-^2^H, ^13^C, ^15^N] PD-L1

*Escherichia coli* BL21 (DE3) cells were transformed with pET-21a (+) plasmid encoding PD-L1 gene (residues Ala18-Tyr134). To obtain uniformly isotopically enriched PD-L1 [U-^13^C, ^15^N], the cells were cultured in M9 Minimal Medium supplied with 3 g of ^13^C-glucose, 1.1 g of ^15^N-NH_4_Cl, 1 cm^3^ of 0.1 mg·cm^–3^ solution of ampicillin, 1 cm^3^ of 1 mg·cm^–3^ thiamine, 1 cm^3^ of 1 mg·cm^–3^ biotin, 1 mmol·dm^–3^ MgSO_4_, 0.3 mmol·dm^–3^ CaCl_2_, grown at 310 K, until OD600 reached 0.8, then induced with 1 mmol·dm^–3^ isopropyl β-d-1-thiogalactopyranoside. They were further grown at 310 K overnight and then harvested by centrifugation at 7500*g* (JA-10 Beckman Coulter) for 15 min at 277 K.

For uniformly isotopically enriched PD-L1 [U-^2^H, ^13^C, ^15^N], the cells were cultured in ^2^H-^13^C-^15^N-enriched medium (*E. coli*-OD2 rich growth media) containing 1 cm^3^ of 0.1 mg·cm^–3^ solution of ampicillin, grown at 310 K, until OD600 reached 0.6, then induced with 1 mmol·dm^–3^ isopropyl β-d-1-thiogalactopyranoside; all reagents were previously dissolved in ^2^H_2_O. The cells were further grown at 310 K overnight and then harvested by centrifugation at 7500*g* (JA-10 Beckman Coulter) for 15 min at 277 K. In all instances, the pellet was suspended, first, in 50 mmol·dm^–3^ Tris-HCl pH 8.0, 200 mmol·dm^–3^ NaCl, 10 mmol·dm^–3^ β-mercaptoethanol, and 10 mmol·dm^–3^ ethylenediaminetetraacetic acid (EDTA) (50 cm^3^ per dm^3^ of culture) and sonicated for 30 s 10 times on ice at 277 K. The suspension was centrifuged at 115,000*g* (Beckman Optima LE-80K Ultracentrifuge) for 40 min and the supernatant discarded. The recovered pellet was resuspended in 50 mmol·dm^–3^ Tris-HCl pH 8.0, 200 mmol·dm^–3^ NaCl, 10 mmol·dm^–3^ β-mercaptoethanol, 6 mol·dm^–3^ guanidinium chloride (25 cm^3^ per dm^3^ of culture) and newly incubated at 277 K overnight under magnetic stirring. Again, the suspension was centrifuged at 115,000*g* (Beckman Optima LE-80K Ultracentrifuge) for 40 min. The pellet was discarded, whereas the supernatant containing the denatured protein solution was diluted in a refolding buffer containing 0.1 mol·dm^–3^ Tris-HCl, pH 8.5, 1 mol·dm^–3^ arginine, 0.25 mmol·dm^–3^ reduced glutathione, and 0.25 mmol·dm^–3^ oxidized glutathione.^73^ The solution was incubated at 277 K under stirring, for 12–18 h, cleared by passing a 0.45 μm filter, and then dialyzed extensively against 10 mmol·dm^–3^ Tris, pH 8.0, 20 mmol·dm^–3^ NaCl. The protein solution was concentrated with an Amicon Stirred Cell and then purified by size exclusion chromatography on HiLoad Superdex 26/60 75pg (GE Healthcare) previously equilibrated in 0.1 mol·dm^–3^ 4-(2-hydroxyethyl)-1-piperazineethanesulfonic acid (HEPES) pH 6.8 and 20 mmol·dm^–3^ NaCl.

### NMR Measurements

Solution NMR experiments for backbone resonance assignment [three-dimensional (3D) HNCA,^74−76^ HNCACB,^77,78^ CBCA(CO)NH,^78,79^ HNCO^74−76^] were performed on [U-^13^C, ^15^N] samples of PD-L1 (at the concentration of 150 μmol·dm^–3^) in the water buffer solution where the protein was more stable [10 mmol·dm^–3^ Tris, pH 8, 20 mmol·dm^–3^ NaCl, 0.1% NaN_3_, protease inhibitors (Roche)]. For 3D HNCACB nonuniform random sampling at 64% and compressed-sensing reconstruction was used.^80^ A 3D HNCA was also recorded at a lower pH [buffer: 20 mmol·dm^–3^ HEPES, pH 6.8, 20 mmol·dm^–3^ NaCl, 0.1% NaN_3_, protease inhibitors (Roche)] to identify a higher number of spin systems and to transfer the protein assignment to buffer conditions closer to those of the anti-PD-L1 fusion protein. All solution spectra were recorded at 298 K on Bruker AVANCE III and AVANCE NEO NMR spectrometers, operating at 1200, 950, and 900 MHz, ^1^H Larmor frequency (28.2, 22.3, and 21.1 T), respectively, equipped with triple-resonance cryo-probes.

Complexes of [U-^15^N], [U-^13^C, ^15^N] or [U-^2^H, ^13^C, ^15^N] PD-L1 with the anti-PD-L1 fusion protein were prepared by adding increasing aliquots of product [10 mg·cm^–3^ (∼50 μmol·dm^–3^)] to the solution of PD-L1 [50 μmol·dm^–3^ in 100 mmol·dm^–3^ HEPES, 20 mmol·dm^–3^ NaCl, pH 6.8] to reach the concentrations of 2.5, 5, and 7.5 μmol·dm^–3^ of anti-PD-L1 fusion protein. Each addition of the anti-PD-L1 fusion protein was monitored by two-dimensional (2D) ^1^H-^15^N SOFAST HMQC spectra.^81^ The excess of unbound PD-L1 was then purified from the complex by HiLoad Superdex 16/60 200pg gel filtration (GF) chromatography and buffer-exchanged to 1 mmol·dm^–3^ HEPES and 4 mmol·dm^–3^ NaCl. The solutions of the complexes (containing ∼10 mg of material) were freeze-dried and the materials used to pack 3.2 mm zirconia thin-wall rotors (open-ended, with bottom and top Vespel caps, Bruker Biospin). The dry samples were then rehydrated by multiple additions of Milli-Q H_2_O until the resolution of the one-dimensional (1D) {^1^H}^13^C CP^82^ (ω_H_ = 70 kHz; ω_C_ = 42 kHz) spectra stopped changing. Silicon plugs (courtesy of Bruker Biospin) placed below the turbine cap were used to close the rotor and preserve hydration. The complex between [U-^2^H, ^13^C, ^15^N] PD-L1 and anti-PD-L1 fusion protein was subsequently transferred in a 1.3 mm zirconia rotor (Bruker Biospin).

A sample of PD-L1 in the presence of a nonbinding antibody (nb-mAb) was also prepared as reference sample. Increasing aliquots of this product [25 mg cm^–3^ (∼170 μmol·dm^–3^)] to reach the concentrations of 12.5 and 25 μmol·dm^–3^ nb-mAb were added to the solution of [U-^13^C, ^15^N] PD-L1 [50 μmol·dm^–3^ in 100 mmol·dm^–3^ HEPES, 20 mmol·dm^–3^ NaCl, pH 6.8]. PD-L1 and nb-mAb were co-lyophilized, and the material (∼13.4 mg) used to fill thick walls 3.2 mm zirconia rotor. Also in this case, the dry material was rehydrated with Milli-Q H_2_O and the spectra acquired.

Another control sample of [U-^13^C, ^15^N] free PD-L1 was prepared by lyophilization in the presence of PEG, and spectra were acquired before and after rehydration, for reference to the SSNMR.

The SSNMR spectra of PD-L1 in the presence of mAbs were collected on a Bruker Avance III spectrometer operating at 800 MHz, ^1^H Larmor frequency (18.8 T, 201.2 MHz ^13^C Larmor frequency), equipped with a Bruker 3.2 mm Efree, and Bruker 1.3 mm NCH probe-heads. The spectra of the free protein were, instead, acquired on a Bruker Avance III 850 MHz, ^1^H Larmor frequency, wide-bore spectrometer (20 T, 213.6 MHz ^13^C Larmor frequency), equipped with a 3.2 mm DVT MAS probe head in triple-resonance mode. The spectra were recorded at 14 and 60 kHz MAS frequencies, for the 3.2 and 1.3 mm rotors, respectively, and the sample temperature was kept at ∼290 K.

Standard ^13^C-detected SSNMR spectra [2D ^15^N-^13^C NCA, ^15^N-^13^C NCO, and ^13^C-^13^C DARR, mixing time 50 ms]^83−87^ were acquired on the samples in 3.2 mm rotors, while ^1^H-detected SSNMR spectra [2D ^15^N-^1^H (H)NH CP-heteronuclear single quantum coherence (HSQC), 3D (H)CANH, 3D (H)CONH, and the ^1^H-^13^C 2D plane of 3D (H)(CA)CB(CA)NH]^88^ were acquired on the sample in 1.3 mm rotor, using the pulse sequences reported in the literature.^89−92^ Experimental details are reported in Tables S1 and S2. For comparison, two-dimensional carbon-detected solution NMR spectra [^13^C-^15^N CON (best-version), CACO and CBCACO]^93,94^ were acquired using a Bruker AVANCE NEO 700 spectrometer equipped with a triple-resonance Cryo-Probe optimized for ^13^C-direct detection, on a sample of free PD-L1 (50 μmol·dm^–3^ in 100 mmol·dm^–3^ MES, pH 6.8, 20 mmol·dm^–3^ NaCl).

All of the spectra were processed with the Bruker TopSpin 3.2 software and analyzed with the program CARA.^95^

## Results

First, we proceeded to an extensive NMR characterization of the isolated PD-L1 ectodomain in solution and in the solid state to evaluate the quality of the spectra and to perform the backbone assignment. Isotopically enriched samples of PD-L1 ectodomain can be expressed in *E. coli*, while the labeling of full-length antibodies is still extremely challenging, although not impossible in principle.

### NMR Characterization of the Isolated PD-L1 Ectodomain

**Figure 2:**
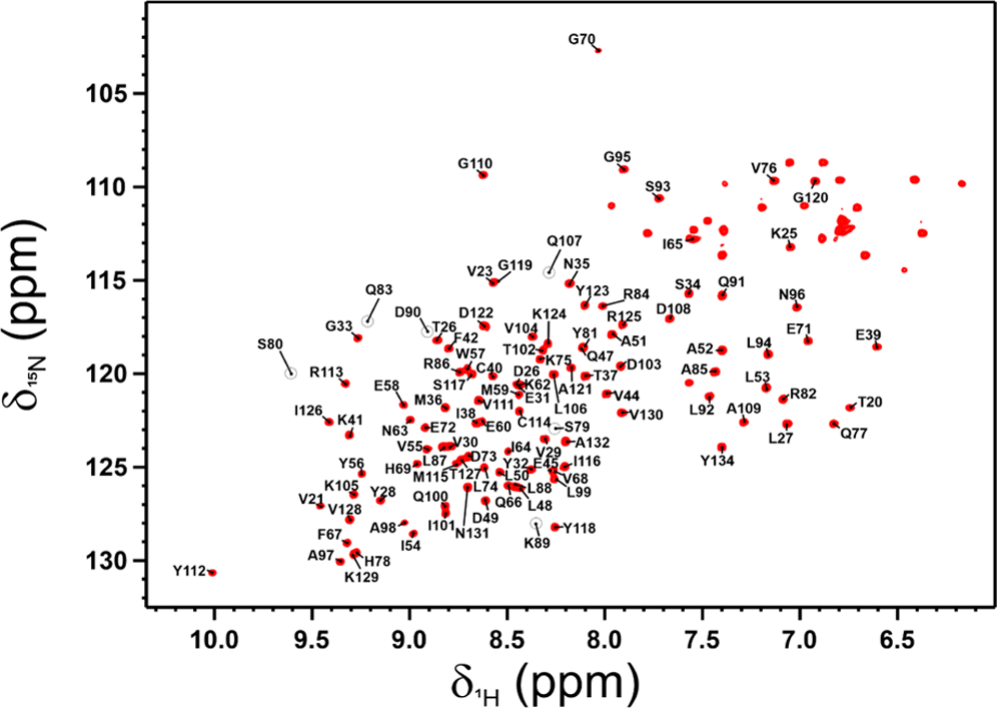
2D ^1^H ^15^N HSQC spectrum of the free PD-L1 in solution [at the concentration of 50 μmol·dm^–3^ in 10 mmol·dm^–3^ Tris, pH 8, 20 mmol·dm^–3^ NaCl, 0.1% NaN_3_, and with protease inhibitors (Roche)] with the assignment of the resonances reported in black. Dashed black circles indicate the missing peaks at pH 8 that conversely were assigned at pH 6.8. The spectrum was acquired on a spectrometer operating at 950 MHz and 298 K.

The 2D ^1^H-^15^N HSQC of free PD-L1 in solution shows sharp and well-resolved signals, as expected for a structured low-molecular-weight protein (∼13.5 kDa). The backbone assignment of free PD-L1 was obtained from the analysis of triple-resonance spectra acquired on samples of [U-^13^C, ^15^N] PD-L1 in solution. All residues but the first three and Asp-61 could be assigned on the spectra (percentage of assignment 97%, Figure 2). In total, 114 signals could be identified and assigned for the free protein in solution. This is, to the best of our knowledge, the only available assignment of PD-L1. The assignment has been deposited on the bmrb under the accession code 51169.

Then, the isolated PD-L1 ectodomain was freeze-dried and the sample was analyzed by SSNMR. As expected for a small protein, the 1D {^1^H}^13^C CP spectrum of the dry material displays broad signals (Figure S1). Also the controlled hydration of the material^41,42^ did not improve the quality and resolution of the spectra in the solid state (Figures S1 and S2).

### NMR Analysis of PD-L1 in the Presence of the Anti-PD-L1 Fusion Protein

Samples of the PD-L1/anti-PD-L1 fusion protein complex were prepared by adding a solution of the product to solutions of the isotopically enriched PD-L1, and the titration was monitored by NMR. The addition of the anti-PD-L1 fusion protein to the solution of [U-^13^C, ^15^N] PD-L1 caused a global decrease in the intensity of the target protein’s signals in the 1D ^1^H and 2D ^1^H-^15^N SOFAST HMQC NMR spectra (Figures S3 and S4). This effect is due to the severe broadening of resonances resulting from the increase of the reorientation correlation time experienced by PD-L1, upon binding to the fusion protein.

Substoichiometric concentrations of the anti-PD-L1 drug were added to the PD-L1 solutions. The large PD-L1/anti-PD-L1 fusion protein complex was then purified from the residual free PD-L1 protein by gel filtration (GF) chromatography and characterized by solution NMR. Only a few signals (Gln/Asn side chains and the C-terminal H^N^), corresponding to atoms that preserve internal mobility after binding to the anti-PD-L1 fusion protein, were observed in the 2D ^1^H-^15^N SOFAST HMQC NMR spectrum acquired after GF (Figure S5), while signals of the free PD-L1 protein were completely disappeared.

**Figure 3:**
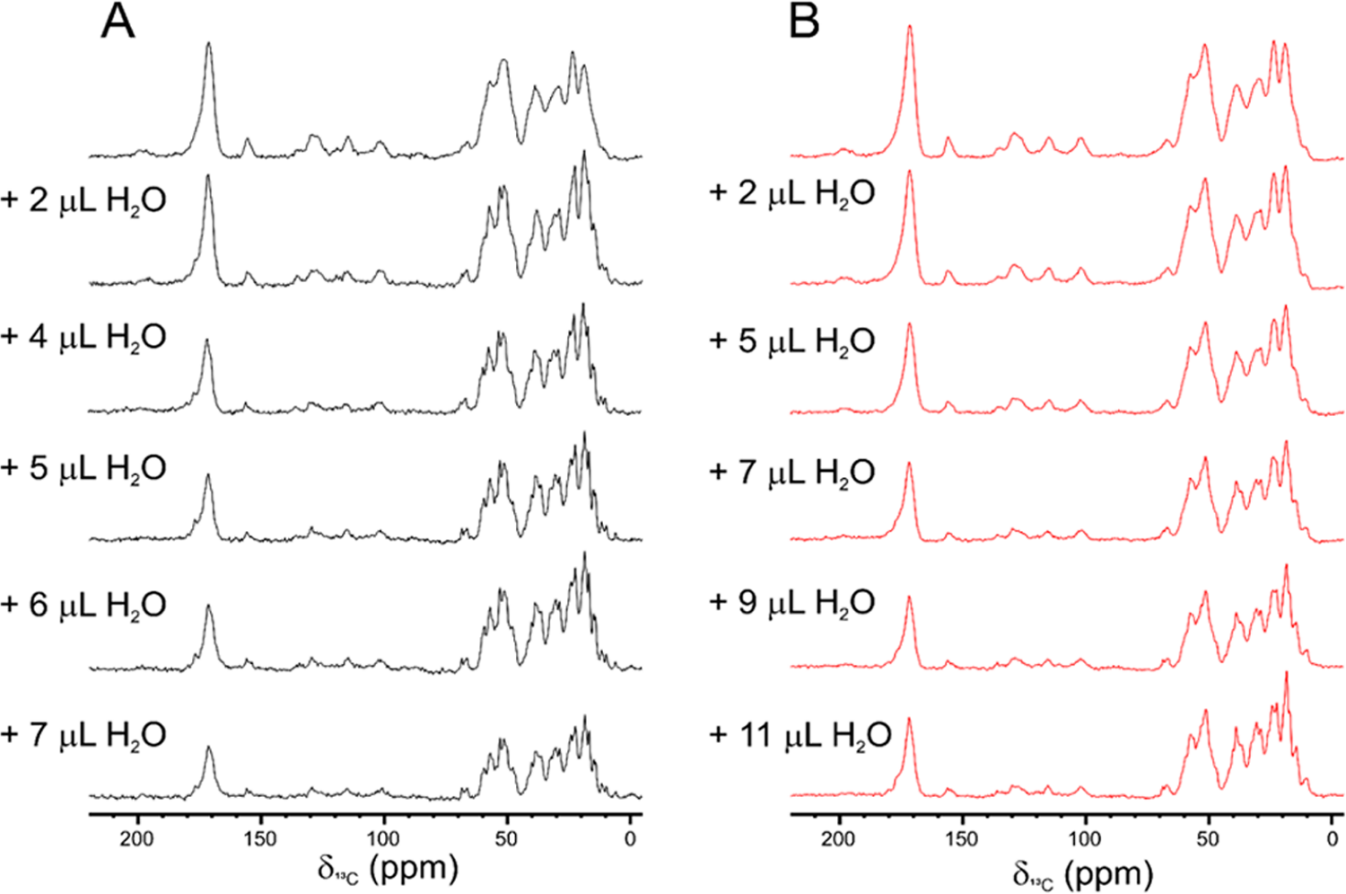
1D {^1^H}^13^C CP SSNMR spectra of freeze-dried [U-^13^C, ^15^N] PD-L1 in complex with the anti-PD-L1 fusion protein (black, A) and in the presence of the nonbinding mAb (red, B). The spectra were recorded on the dried materials and after the addition of increasing amounts of Milli-Q H_2_O. Spectra were acquired on a spectrometer operating at 800 MHz (^1^H Larmor frequency) with a MAS of 14 kHz and a temperature of ∼290 K.

Then, the PD-L1/anti-PD-L1 fusion protein complex was freeze-dried and analyzed by SSNMR. The 1D {^1^H}^13^C CP spectrum collected on the freeze-dried sample was of poor quality. However, the stepwise hydration of the material leads to a significant improvement in quality and resolution of the spectrum (Figure 3A).

**Figure 4:**
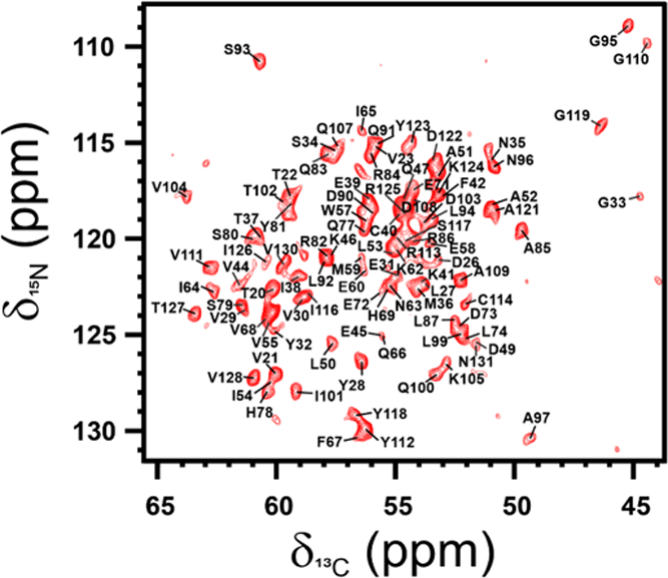
2D ^15^N ^13^C NCA spectrum of [U-^13^C, ^15^N] PD-L1 in complex with the anti-PD-L1 fusion protein. The assignment of the resonances is reported in black. The spectrum was acquired on a spectrometer operating at 800 MHz (^1^H Larmor frequency) with a MAS of 14 kHz and a temperature of ∼290 K.

**Figure 5:**
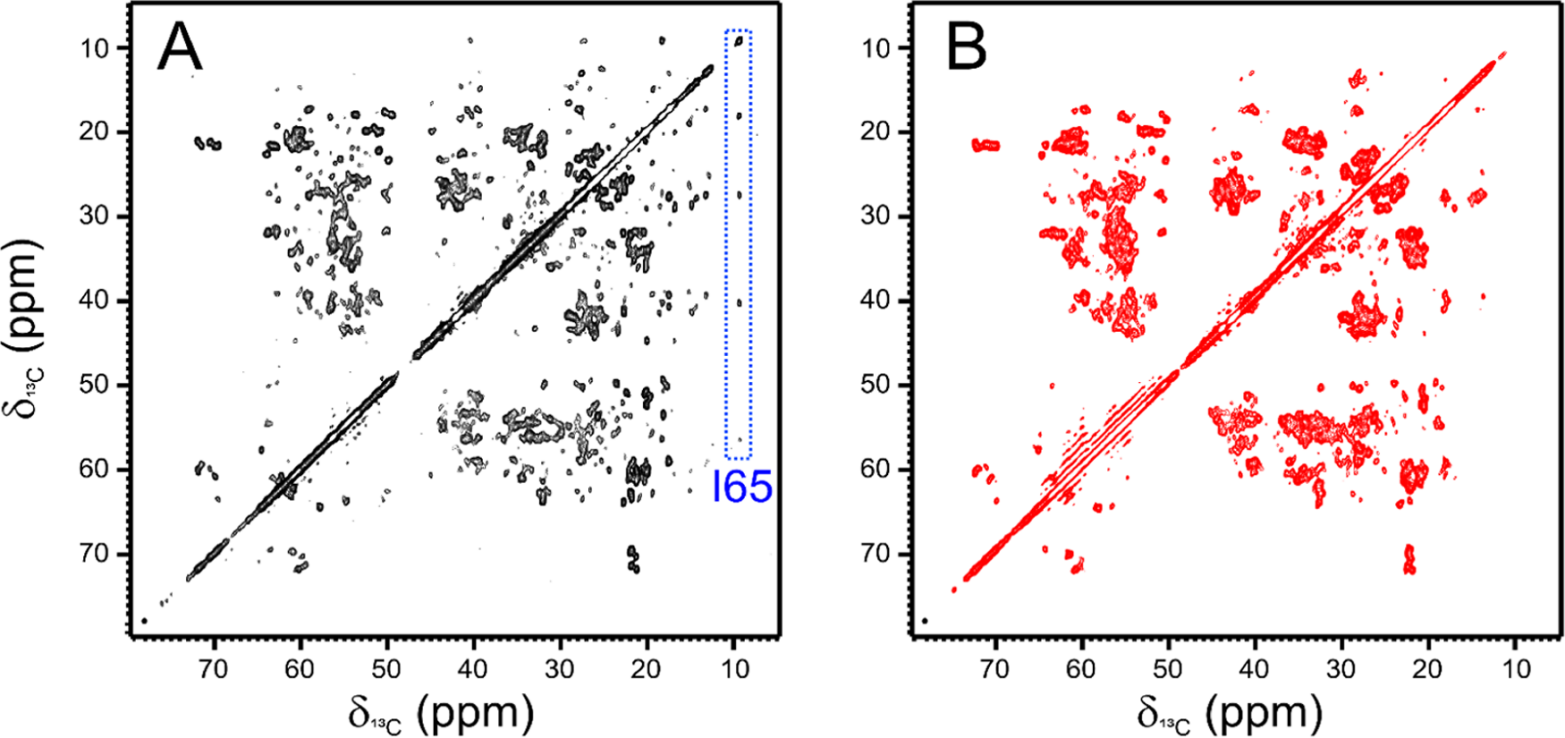
2D ^13^C-^13^C DARR spectra acquired on rehydrated samples of freeze-dried complex of [U-^13^C, ^15^N] PD-L1 with the anti-PD-L1 fusion protein (black, A) and of the mixture of [U-^13^C, ^15^N] PD-L1 with the nonbinding mAb (red, B). The assignment of I65 side chain is indicated by a blue box. Spectra were acquired on a spectrometer operating at 800 MHz (^1^H Larmor frequency) with a MAS of 14 kHz and a temperature of ∼290 K.

Hetero- and homonuclear correlation spectra were recorded on the rehydrated sample (Figure S6) and used for resonance assignment. The assignment of the 2D ^15^N ^13^C NCA spectrum (Figure 4) was obtained starting from the data collected in solution on the isolated PD-L1 and complemented by the analysis of the 2D ^15^N ^13^C NCO and ^13^C-^13^C DARR (Figure 5A) spectra of the complex which allowed us, at the same time, to obtain side-chain assignments. First, the assignment of free PD-L1 in solution was superimposed on the 2D ^15^N ^13^C NCA spectrum (Figure S7A,B). The assignment was then matched to the closest signals in the spectrum by identifying the Cα frequencies of the neighboring signals also in the 2D ^13^C-^13^C DARR spectrum (Figure S7C). The pattern of carbon resonances correlated to the Cα frequencies in the 2D ^13^C-^13^C DARR spectrum allowed us to identify the spin systems characteristic of each residue type and distinguish among possible ambiguities. The resolution of 2D ^15^N ^13^C NCO was lower with respect to the other spectra; however, some signals in the 2D ^15^N ^13^C NCO were helpful in confirming the ^15^N chemical shift values of some residues obtained from the 2D ^15^N ^13^C NCA spectrum.

Finally, a total of 99 spin systems could be identified and assigned in ^13^C-detected spectra. Interestingly, in addition to the three signals missing in solution NMR spectra, the signals of other residues located in flexible loops of PD-L1 (K25, L48, Q66, G70, L74-V76, K89, M115, G120, A132-Y134) are missing in the SSNMR spectra of the complex.

**Figure 6:**
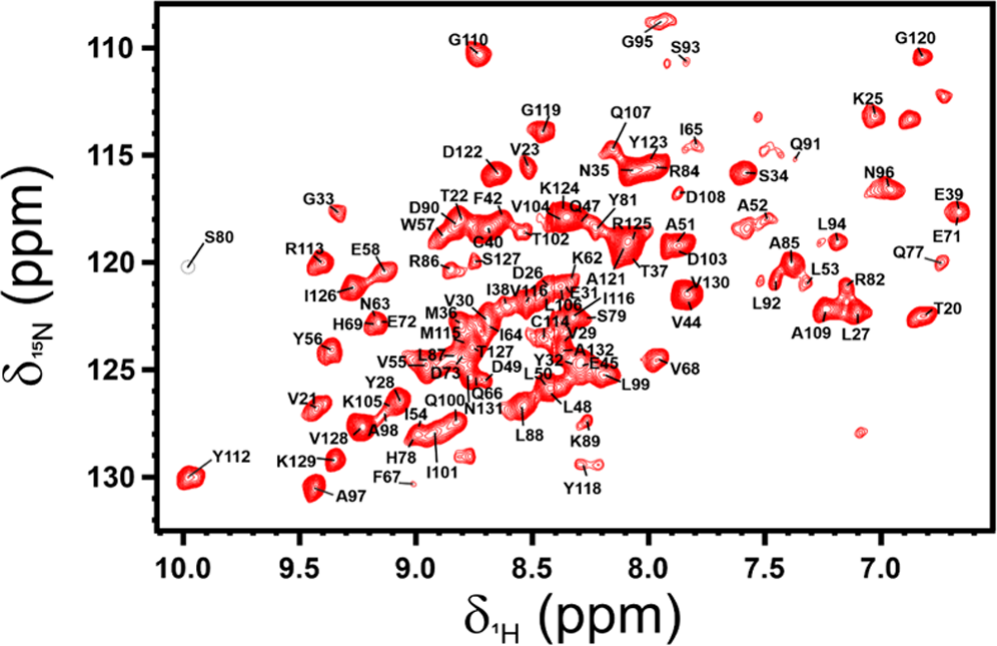
2D ^15^N ^1^H (H)NH CP-HSQC spectrum of [U-^2^H, ^13^C, ^15^N] PD-L1 in complex with the anti-PD-L1 fusion protein. The assigned resonances are reported in black. The spectrum was acquired on the rehydrated freeze-dried material, using a spectrometer operating at 800 MHz (^1^H Larmor frequency) with a MAS of 60 kHz and a temperature of ∼290 K.

To improve the assignment of the resonances and the quality of the chemical shift mapping, a set of ^1^H-detected spectra was also acquired on a sample of [U-^2^H,^13^C, ^15^N] PD-L1 in complex with the anti-PD-L1 fusion protein, prepared under the same experimental conditions of the previously described complex ([U-^13^C, ^15^N] PD-L1/anti-PD-L1 drug). The sample was then transferred in a 1.3 mm rotor. The 2D ^15^N-^1^H (H)NH CP-HSQC spectrum of the PD-L1/anti-PD-L1 fusion protein complex is of high quality (Figure 6). Also in this case, the assignment of the SSNMR spectrum was obtained starting from the available assignment of the free protein in solution and confirmed by the analysis of 3D spectra [(H)CANH, (H)CONH, 2D ^13^C-^1^H plane of (H)(CA)CBNH]. Also in the ^1^H-detected spectra, some signals of residues belonging to flexible loops of PD-L1 (K41, K46, M59-D61, Q66, G70, L74-V76, Q83, L106, Y134) are missing. Summarizing, a total of 99 spin systems could be identified and assigned also in the ^1^H-detected spectra. Interestingly, in the solid state, some signals could be identified in the ^13^C-detected spectra, while others in the ^1^H-detected spectra.

### Chemical Shift Perturbation (CSP) Can Map the Binding Regions of PD-L1

**Figure 7:**
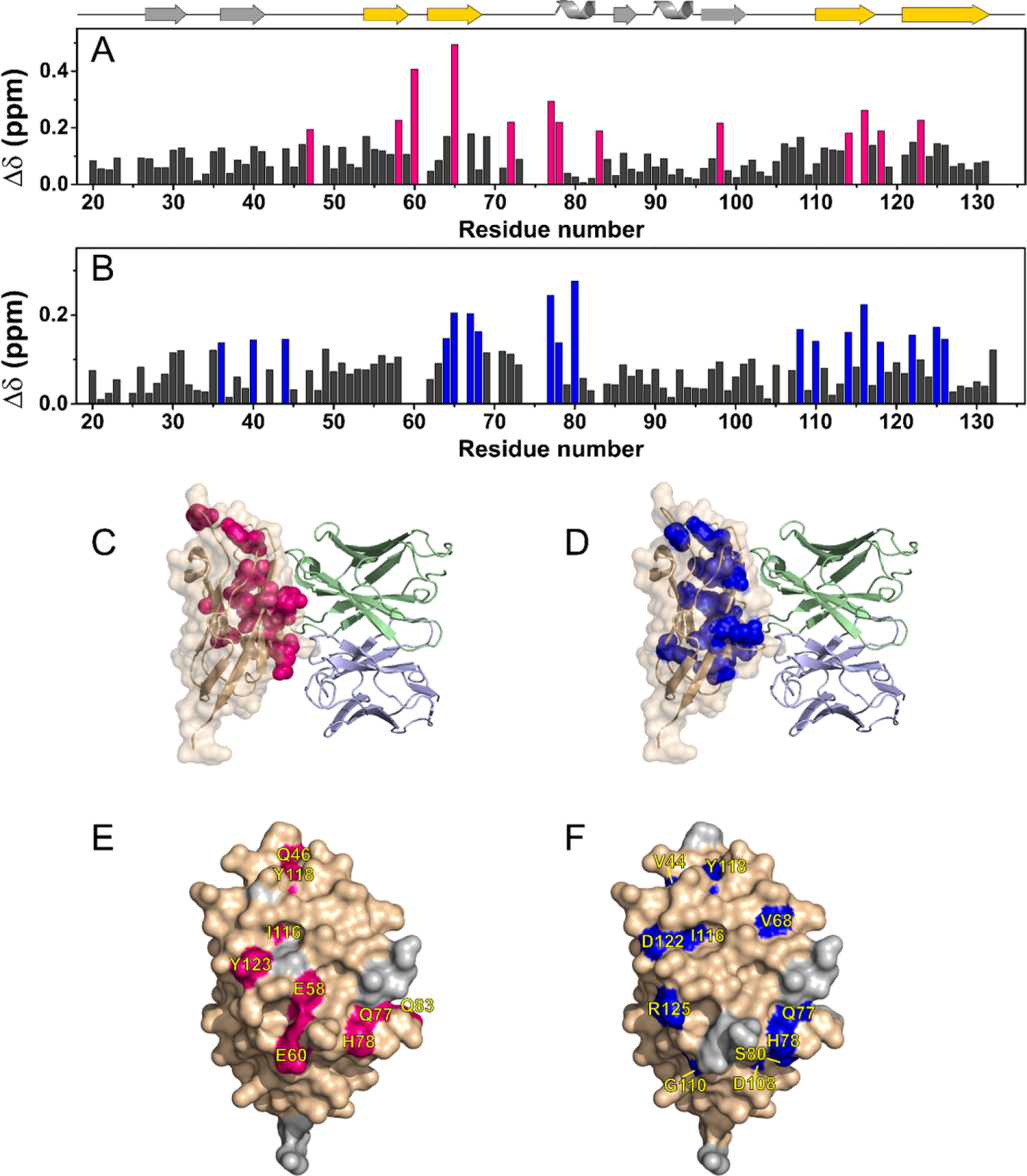
Chemical shift perturbation (CSP) of free PD-L1 in solution with respect to rehydrated freeze-dried PD-L1/anti-PD-L1 fusion protein complex in the solid state, evaluated according to the formula (A)  and (B) . The residues experiencing the largest variations (> mean + σ) have been highlighted in magenta and blue, respectively. The secondary structure representation is reported on the top of the plot. The β-strands facing Avelumab in the structure of the complex are highlighted in yellow. (C, D) CSP mapping (on the structure with PDB code: 5GRJ) with all of the residues experiencing the largest perturbations colored in magenta and blue, respectively. (E, F) Interacting surface of PD-L1 in 5GRJ with only the solvent-exposed residues experiencing the largest CSP highlighted in magenta and blue, respectively. The solvent-exposed residues are labeled in yellow. The residues missing in the 2D ^15^N ^13^C NCA and in the 2D ^15^N ^1^H (H)NH CP-HSQC spectra are colored in light gray.

The availability of protein assignment for the isolated PD-L1 ectodomain in solution and for the same protein in complex with the anti-PD-L1 fusion protein in the solid state allows for the analysis of the chemical shift perturbation (CSP). The CSP of ^13^Cα/^15^N and ^1^H/^15^N resonances was calculated from the assignment of ^13^C- and ^1^H-detected SSNMR spectra, respectively, using the assignment of the isolated [U-^2^H, ^13^C, ^15^N] PD-L1 obtained in solution as reference. Although all residues experience a chemical shift variation moving from solution to solid-state experiments,^34^ those experiencing the largest chemical shift variations (Q47, E58, E60, I65, E72, Q77, H78, Q83, A93, C114, I116, Y118, and Y123 according to ^13^Cα/^15^N chemical shift values; M36, C40, V44, I64, I65, F67, V68, Q77, H78, S80, D108, G110, C114, I116, Y118, D122, R125, and I126 according to ^1^H/^15^N chemical shift values) are located on PD-L1 β-sheets and form a large interaction surface (Figure 7).

The CSP values were also analyzed using different thresholds obtained from the iterative procedure proposed by Schumann and co-workers.^96^ Interestingly, this analysis showed that residues below the new calculated threshold are located in regions noninteracting with the anti-PDL-1 fusion protein (see the Supporting Information for more details, Figure S8).

### Comment about Spectral Quality

**Figure 8:**
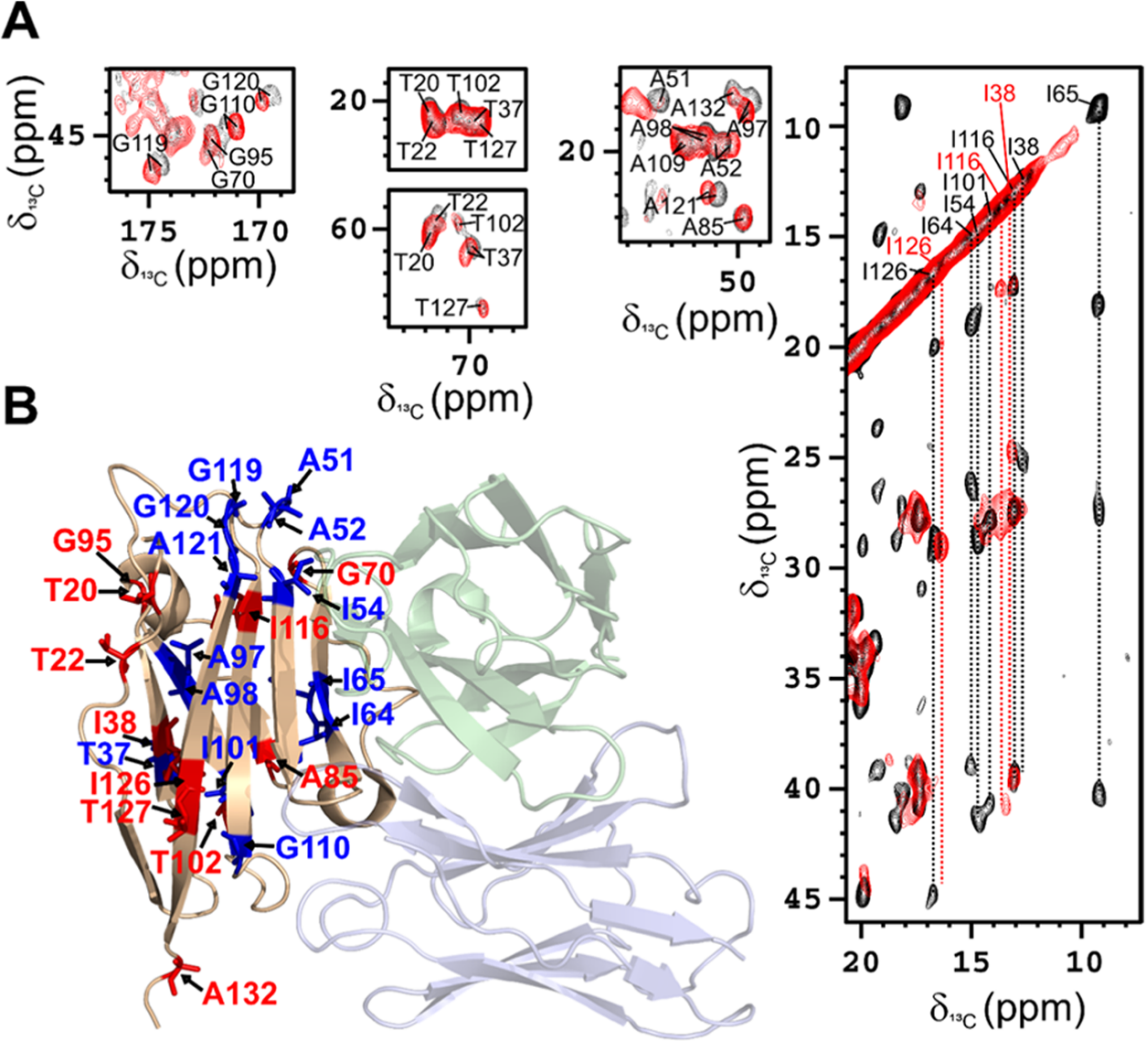
(A) Selected regions (C/Cα of Gly, Cβ/Cγ_2_ and Cβ/Cα of Thr, Cα/Cβ of Ala, Cδ_1_/C_ali_ of Ile) of 2D ^13^C-^13^C DARR spectra acquired on samples of rehydrated freeze-dried complex of [U-^13^C, ^15^N] PD-L1 with the anti-PD-L1 fusion protein PD-L1 (black) and rehydrated freeze-dried mixture of [U-^13^C, ^15^N] PD-L1 with nonbinding mAb (red). The assignment of the two spectra is reported. In the Ile region of PD-L1/nb-mAb spectrum, some signals are missing. The signals of the assigned Ile in this region are labeled in red. (B) Cartoon representation of the structure of PD-L1 in complex with Avelumab-scFv (PDB code: 5GRJ), with highlighted in blue the residues experiencing the largest shift and in red the nonshifting/nonpresent residues in the spectra of PD-L1/nb-mAb with respect to the spectra of PD-L1 in the presence of anti-PD-L1 fusion protein.

To confirm that the observed improvement in quality of the solid-state spectra of PD-L1 was due to its binding to the anti-PD-L1 fusion protein, the target was titrated with a noninteracting monoclonal antibody (nb-mAb). As expected, also at high concentrations (PD-L1: nb-mAb, 1:0.5 molar ratio, Figure S9), this antibody does not affect the signals of PD-L1 in a 2D ^1^H-^15^N SOFAST HMQC NMR spectrum. Then, the PD-L1/nb-mAb mixture was freeze-dried and analyzed by SSNMR in a 3.2 mm rotor. The experiments recorded on the sample show that in the presence of the nonbinding mAb, the stepwise rehydration does not improve sizably the quality and resolution of the solid-state spectra (Figures 3B and 5B). However, in some regions of this DARR spectrum, the signals are sufficiently resolved to be assigned and compared with those present in the 2D DARR spectrum recorded on the PD-L1/anti-PD-L1 fusion protein complex (Figure 8). The analysis of the two spectra allowed us to evaluate the occurrence of a meaningful chemical shift perturbation for some signals. Most of the signals experiencing the largest shift are indeed located on PD-L1 β-sheets that form the binding surface for anti-PD-L1 fusion protein. Conversely, the signals experiencing negligible effects are located on the opposite face of the PD-L1 protein. In this respect, it is interesting to point out that the signals of Ile54, Ile64, and Ile65, placed on the binding interface, are missing in the DARR spectrum of PD-L1 in the presence of nonbinding mAb, while they are present in the DARR spectrum of the PD-L1/anti-PD-L1 fusion protein complex. The appearance of these signals is consistent with a unique and more rigid conformation of the related residues due to the interaction with the anti-PD-L1 fusion protein.

## Discussion

The last advances in antibody engineering have led to the development of complex fused biologics with multispecific activity and increased structural complexity. Understanding such a structural complexity and how it impacts the function of a biotherapeutic is, on the one hand, not a trivial task, but, on the other hand, it is of paramount importance during drug development because it is strictly linked to the QbD concept. Indeed, detailed product knowledge is instrumental to the production of safer and more effective drugs and to improve process control strategies.

The epitope mapping on a target can provide the structural information needed to understand the mechanism of action of biologics by supporting structure–activity relationship (SAR) studies, that are critical during pharmaceutical development. SAR can indeed be used to explain the different ways in which a ligand interacts with a receptor: this, in turn, can be used to optimize the physicochemical and functional properties of a biotherapeutic (e.g., solubility, potency, pharmacokinetics, etc.) and can support the design of mutants with larger interacting surfaces and affinities or capable of binding mutated targets.

The results here reported prove that a detailed characterization of the binding to the target of very large and flexible biologics can be achieved by integrating solution and solid-state NMR experiments. The epitope mapping on PD-L1 obtained by this NMR approach nicely matches with the interacting surface previously observed in the X-ray structure of the PD-L1 in complex with Avelumab-scFv (PDB code: 5GRJ),^97^ another anti-PD-L1 mAb that shares with the tested fusion protein the same Fab sequence (only three amino acids are mutated). Most of the residues experiencing the largest effects are hydrophobic amino acids: aromatic and aliphatic residues forming a wide hydrophobic patch on PD-L1 that is targeted by the anti-PD-L1 fusion protein. At the same time, residue R125 of PD-L1 that in the crystallographic complex^97^ is close to residue S95 of Avelumab, as well as E58 that is involved in hydrogen bonding with residue Y52 of mAb experience a large chemical shift variation in the presence of our tested anti-PD-L1 fusion protein.

An additional aspect that should be considered is the importance of the characterization of a protein structure *per se* and not necessarily when the molecule is bound to its target. Indeed, the higher-order structure (HOS) of a protein—intended as secondary, tertiary, and quaternary structures—is a fingerprint covering structural quality attributes potentially linked to the function of a biologic that is constantly monitored during its development. Unwanted perturbations of the folding introduced during the manufacturing process or formulation optimization may in fact lead, for example, to loss of function and/or immunogenicity. The dependence of the binding mechanism on the structural features of the interacting proteins suggests the use of our epitope mapping approach in HOS comparative studies, as the solid-state NMR spectra of the complex allow us to map the fingerprint of a biologic “left” on the target. The chemical shift perturbation (CSP) experienced by the target in the complex is sensitive to the HOS of the antibody—or at least of its binding domain—and it can be used as an “indirect” measure of the ligand structure.

Overall, this approach opens new ways to monitor HOS during pharmaceutical development, allowing us to focus on the structural alterations that may affect target recognition and binding affinity, thus linking HOS assessment to the drug mechanism of action.

The experimental protocol used here to prepare the sample is simple and every step is easily controlled. The methodology does not require the isotopic enrichment of the biological drug, which is usually expressed in eukaryotic cells and where the labeling is highly expensive, although feasible. Conversely, targets can often be obtained in *E. coli* expression system where the labeling is easy, inexpensive, and with high yields.

## Supporting Information Available

1D {^1^H}^13^C, 2D ^13^C-^13^C, and 2D ^15^N-^13^C solid-state NMR spectra; 2D ^1^H-^15^N solution NMR spectra; details of assignment and CSP; acquisition parameters for SSNMR spectra; and assignment tables (PDF)

## Notes

NMR assignment in solution of PD-L1 ectodomain (residues Ala18-Tyr134) generated during the current study is available in the BMRB database under the accession code: 51169. The raw data are available at https://zenodo.org under the DOI: 10.5281/zenodo.6363169.
