# Supplementary material for: Epitope Mapping and Binding Assessment by Solid-State NMR Provide a Way for the Development of Biologics under the Quality by Design Paradigm
Source: J Am Chem Soc. 2022 May 26;144(22):10006–16. doi: 10.1021/jacs.2c03232 (PMC9185746; doi:10.1021/jacs.2c03232)
Supplement: Supplementary file 1 — ja2c03232_si_001.pdf [file ja2c03232_si_001.pdf]

Epitope mapping and binding assessment by solid-state NMR provide a way for development of biologics under the Quality by Design paradigm

SUPPLEMENTARY INFORMATION

Domenico Rizzo[1,2,§], Linda Cerofolini[1,3,§], Stefano Giuntini[1,2], Luisa Iozzino[4], Carlo Pergola[4], Francesca Sacco,[1,4] Angelo Palmese[4], Enrico Ravera[1,2,3], Claudio Luchinat[1,2,3,*], Fabio Baroni[4,*], Marco Fragai[1,2,3,*]

1. Magnetic Resonance Center (CERM), University of Florence, Via L. Sacconi 6, 50019 Sesto Fiorentino, Italy.
2. Department of Chemistry "Ugo Schiff", University of Florence, Via della Lastruccia 3, 50019, Sesto Fiorentino, Italy.
3. Consorzio Interuniversitario Risonanze Magnetiche di Metalloproteine (CIRMMP) Via L. Sacconi 6, 50019 Sesto Fiorentino, Italy.
4. Analytical Development Biotech Department, Merck Serono S.p.a, Via Luigi Einaudi, 11, 00012, Guidonia, RM, Italy; an affiliate of Merck KGaA.

*Corresponding authors;

luchinat@cerm.unifi.it

fabio.baroni@merckgroup.com

fragai@cerm.unifi.it

[§]D.R. and L.C. contributed equally to this paper

INDEX

Figure S1 .... **Pag. S3**

Figure S2 .... **Pag. S4**

Figure S3 .... **Pag. S5**

Figure S4 .... **Pag. S6**

Figure S5 .... **Pag. S7**

Figure S6 .... **Pag. S8**

Figure S7.. **Pag. S9**

Figure S8.. **Pag. S10**

Figure S9.. **Pag. S11**

Table S1 .... **Pag. S12**

Table S2 .... **Pag. S12**

Table S3 .... **Pag. S13**

Table S4 .... **Pag. S15**

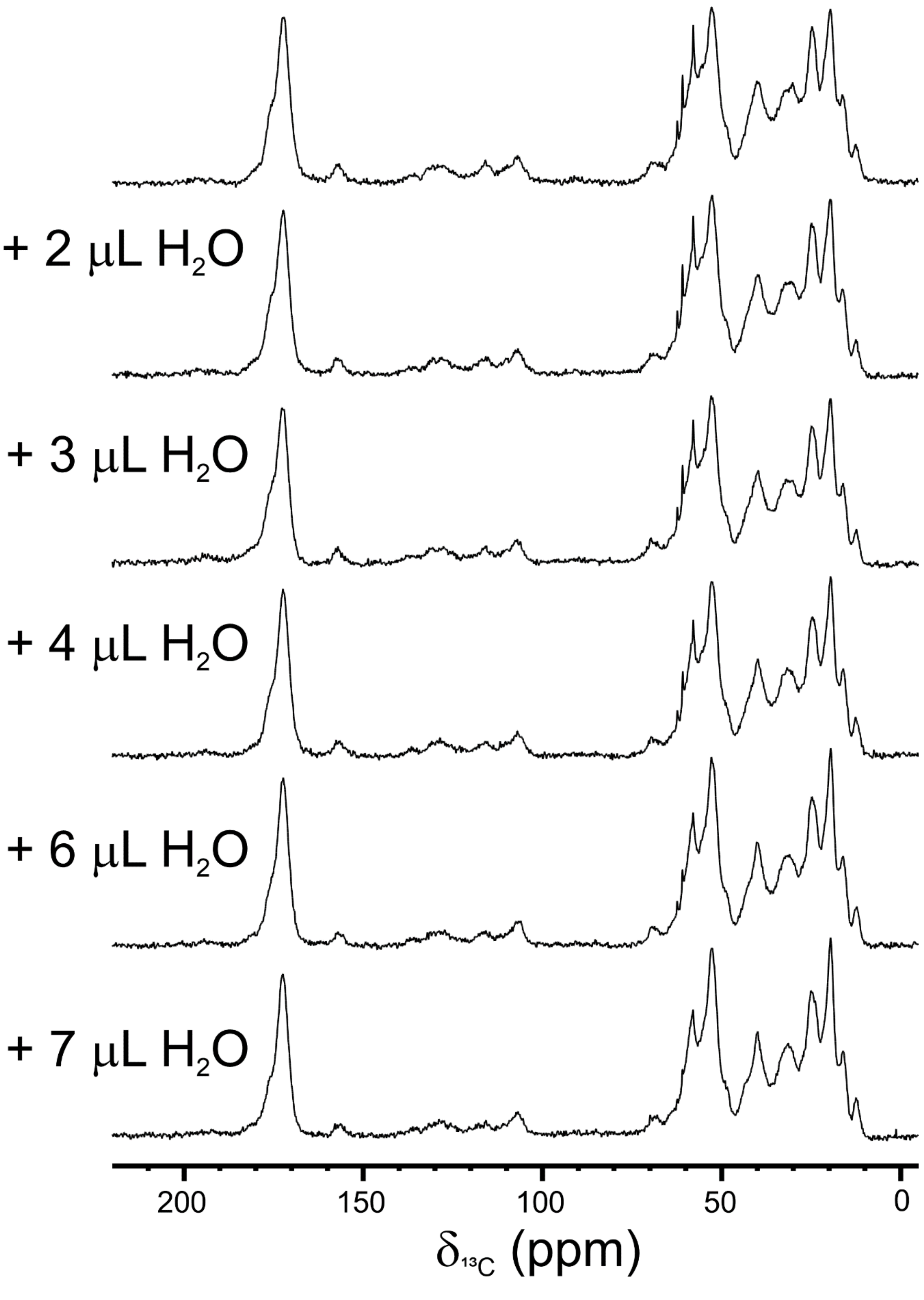

Figure S1. 1D {^{1}H}^{13}C CP SSNMR spectra of free freeze-dried [U-^{13}C, ^{15}N] PD-L1 before and after addition of increasing amount of MilliQ H_2O. The spectra were acquired on a spectrometer operating at 850 MHz ([1]H Larmor frequency) with MAS of 14 kHz and temperature of ~ 290 K.

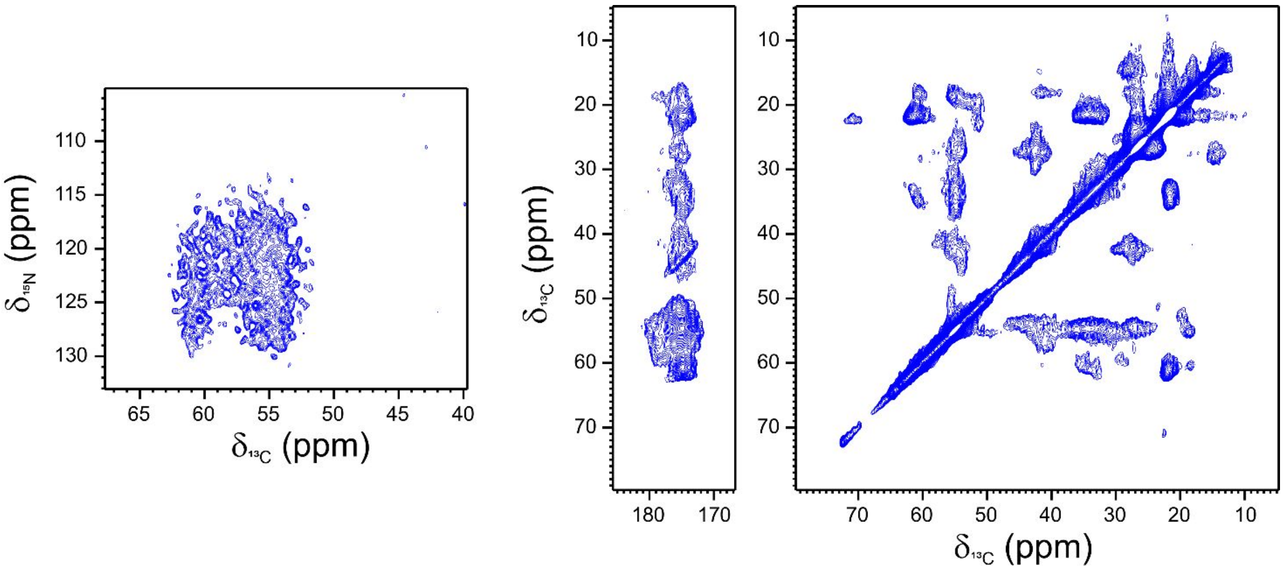

Figure S2. 2D ^{15}N ^{13}C NCA (left) and ^{13}C-^{13}C DARR (right) acquired after re-hydration on a sample of free freeze-dried [U-^{13}C, ^{15}N] PD-L1. The spectra were acquired on a spectrometer operating at 850 MHz (^{1}H Larmor frequency) with MAS of 14 kHz and temperature of ~ 290 K. The spectra have been now processed with QSIN window function and SSB=2.

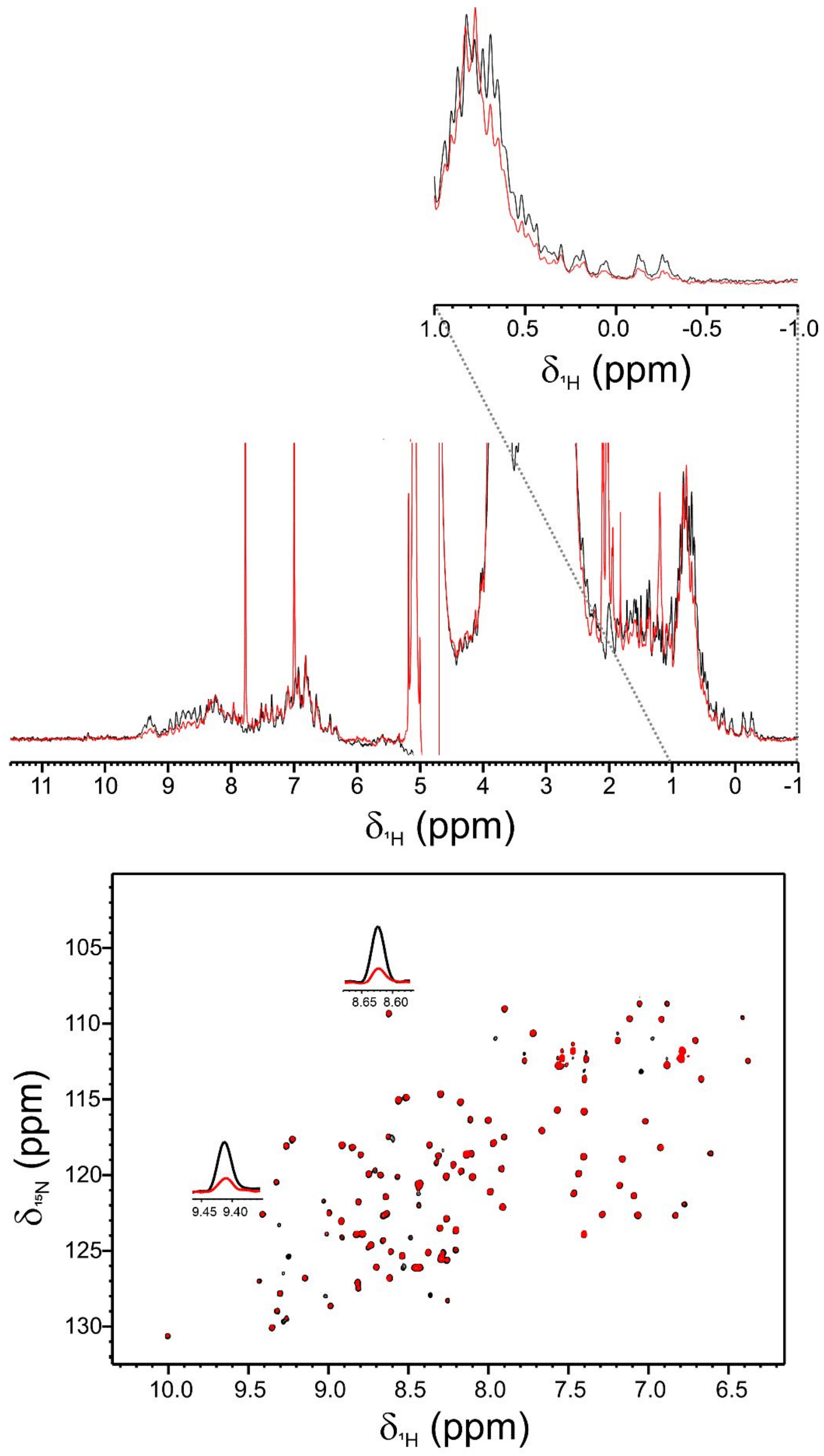

Figure S3. 1D ^{1}H (top) and 2D ^{1}H-^{15}N so-FAST HMQC (bottom) NMR spectra of PD-L1 in the presence of sub-stoichiometric amount of the anti-PD-L1 fusion protein (red) with respect to free PD-L1 (black). Spectra were acquired at 298 K on a spectrometer operating at 950 MHz (^{1}H Larmor frequency). The 1D projections of two signals are displayed in the 2D spectra to show their different intensity.

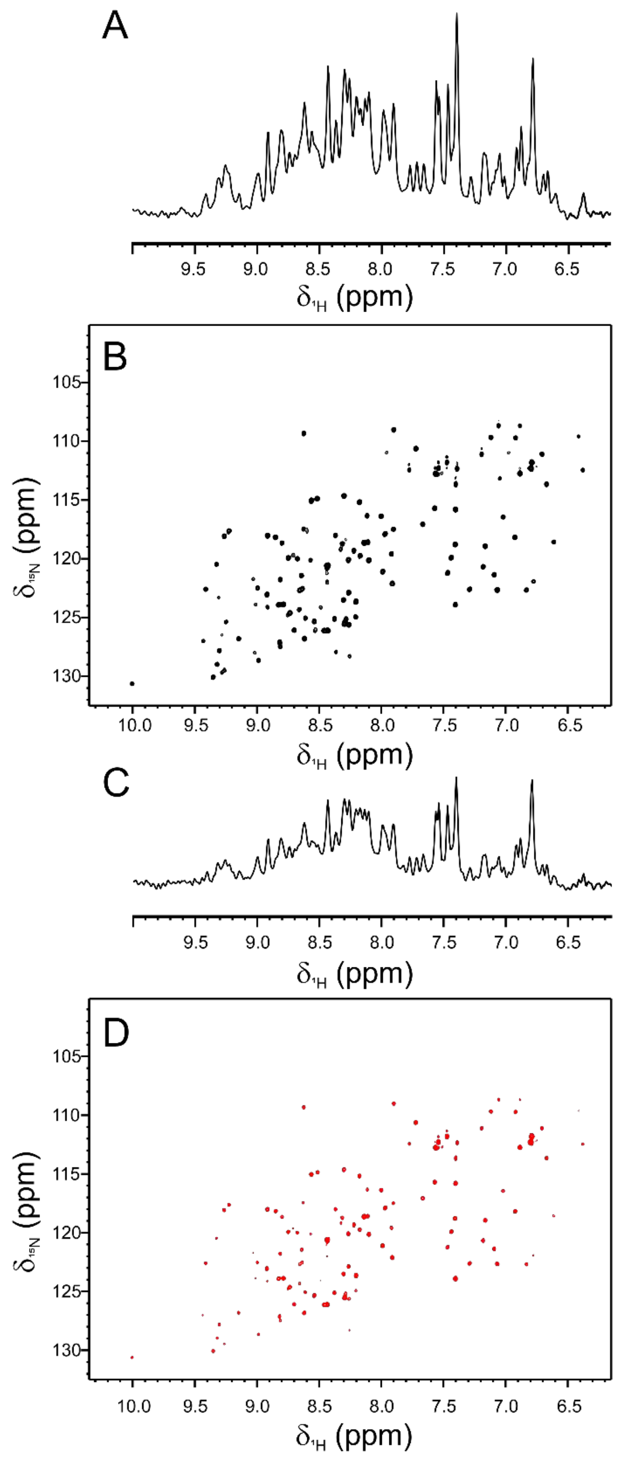

Figure S4. First FID (A, C) and 2D ^{1}H-^{15}N so-FAST HMQC NMR complete spectra (B, D) of PD-L1 acquired in the titration with anti-PD-L1 fusion protein, in the absence (A,B) and in the presence (C,D) of anti-PD-L1 fusion protein in sub-stoichiometric amount with respect to free PD-L1. Spectra were acquired at 298 K on a spectrometer operating at 950 MHz (^{1}H Larmor frequency).

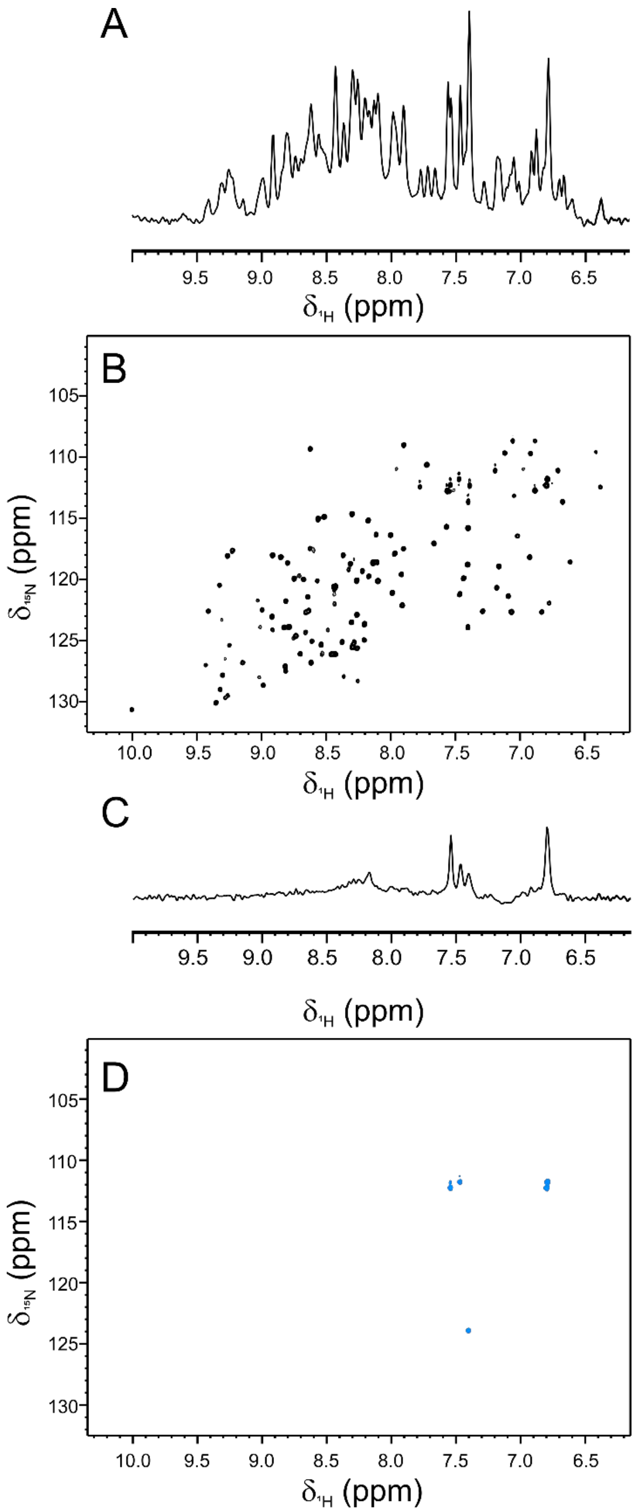

Figure S5. First FID (A, C) and 2D ^{1}H-^{15}N so-FAST HMQC NMR complete spectra (B, D) of free PD-L1 (A, B) and od PD-L1 in complex with the anti-PD-L1 fusion protein acquired after gel-filtration to remove the excess of unbound PD-L1. Spectra were acquired at 298 K on a spectrometer operating at 950 MHz (^{1}H Larmor frequency).

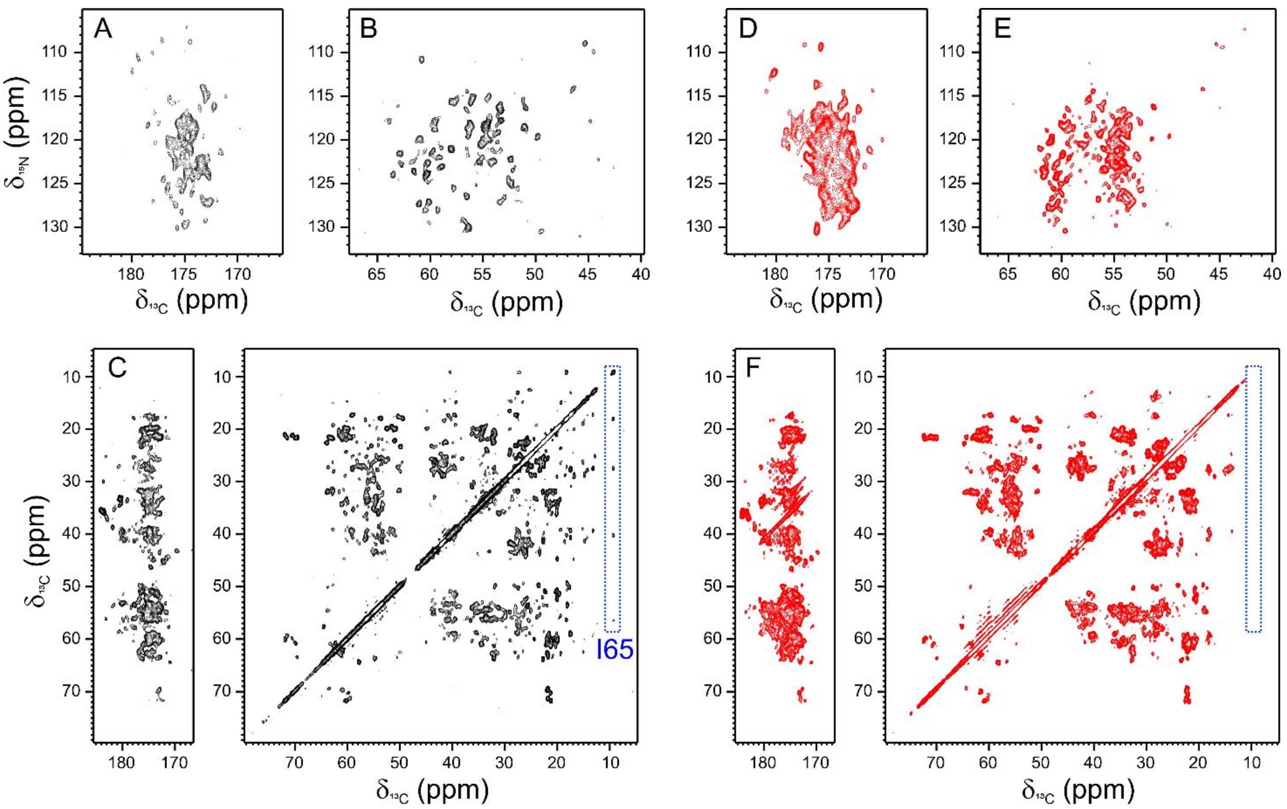

Figure S6. 2D ^{15}N ^{13}C NCO (A, D), ^{15}N ^{13}C NCA (B, E) and ^{13}C-^{13}C DARR (C, F) spectra acquired after re-hydration on samples of freeze-dried complex of [U-^{13}C, ^{15}N] PD-L1 with the anti-PD-L1 fusion protein (black) and freeze-dried mixture of [U-^{13}C, ^{15}N] PD-L1 with non-binding mAb (red). The assignment of I65 sidechain is indicated in the figure by a blue box. Spectra were acquired on a spectrometer operating at 800 MHz (^{1}H Larmor frequency) with MAS of 14 kHz and temperature of ~ 290 K.

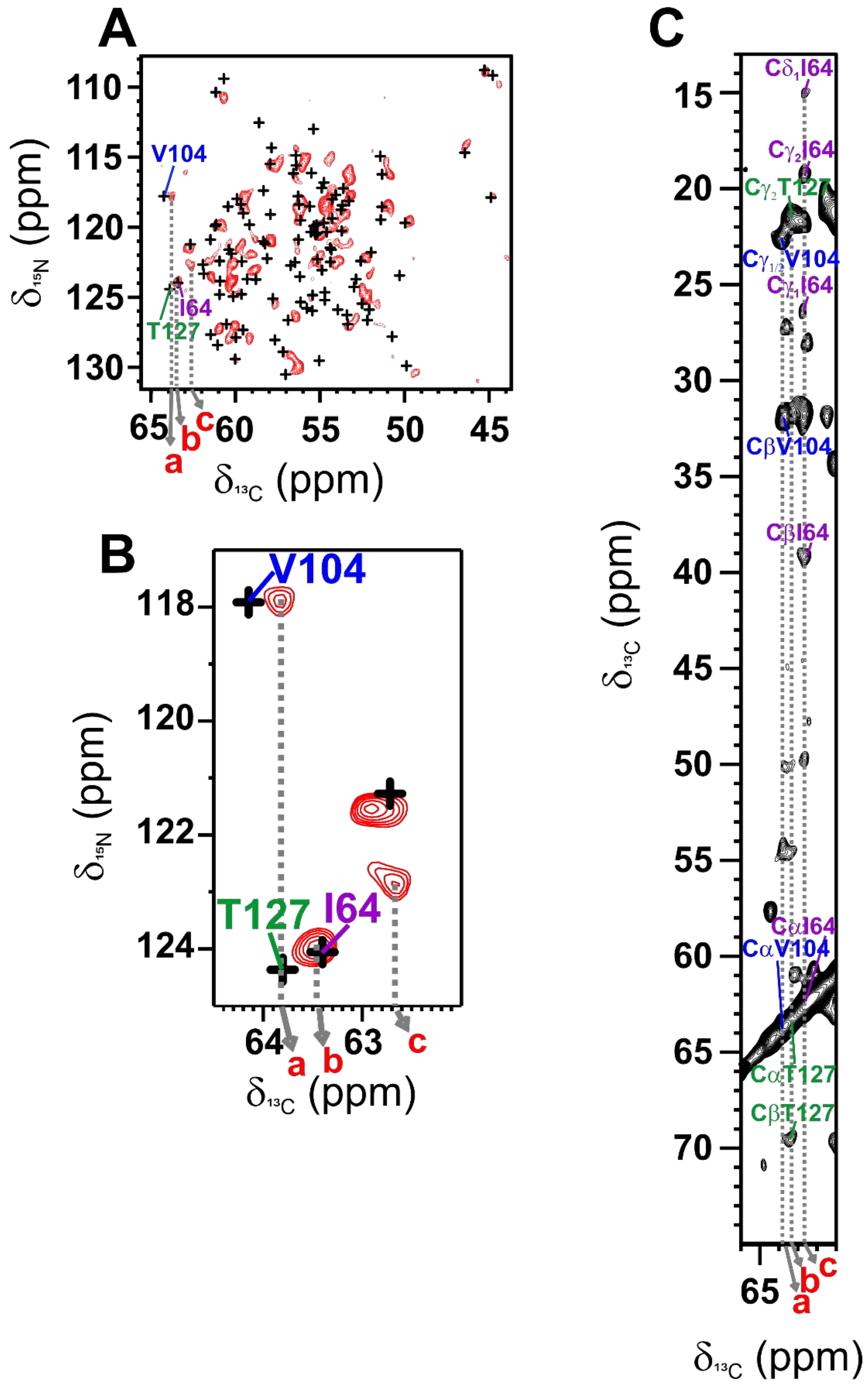

Figure S7. A) Super-imposition of the assignment of free PD-L1 in solution (black cross) on the 2D ^{15}N ^{13}C NCA spectrum of PD-L1 in complex with the anti-PD-L1 fusion protein. The assignment was then matched to the closest signals in the spectrum by identifying the Cα frequencies of the neighboring signals (a, b, c). B) the Cα frequencies were matched also on the 2D ^{13}C-^{13}C DARR spectrum. The pattern of carbon resonances correlated to the Cα frequencies in the 2D ^{13}C-^{13}C DARR spectrum allowed to identify the spin systems characteristic of each residue type and distinguish among possible ambiguities.

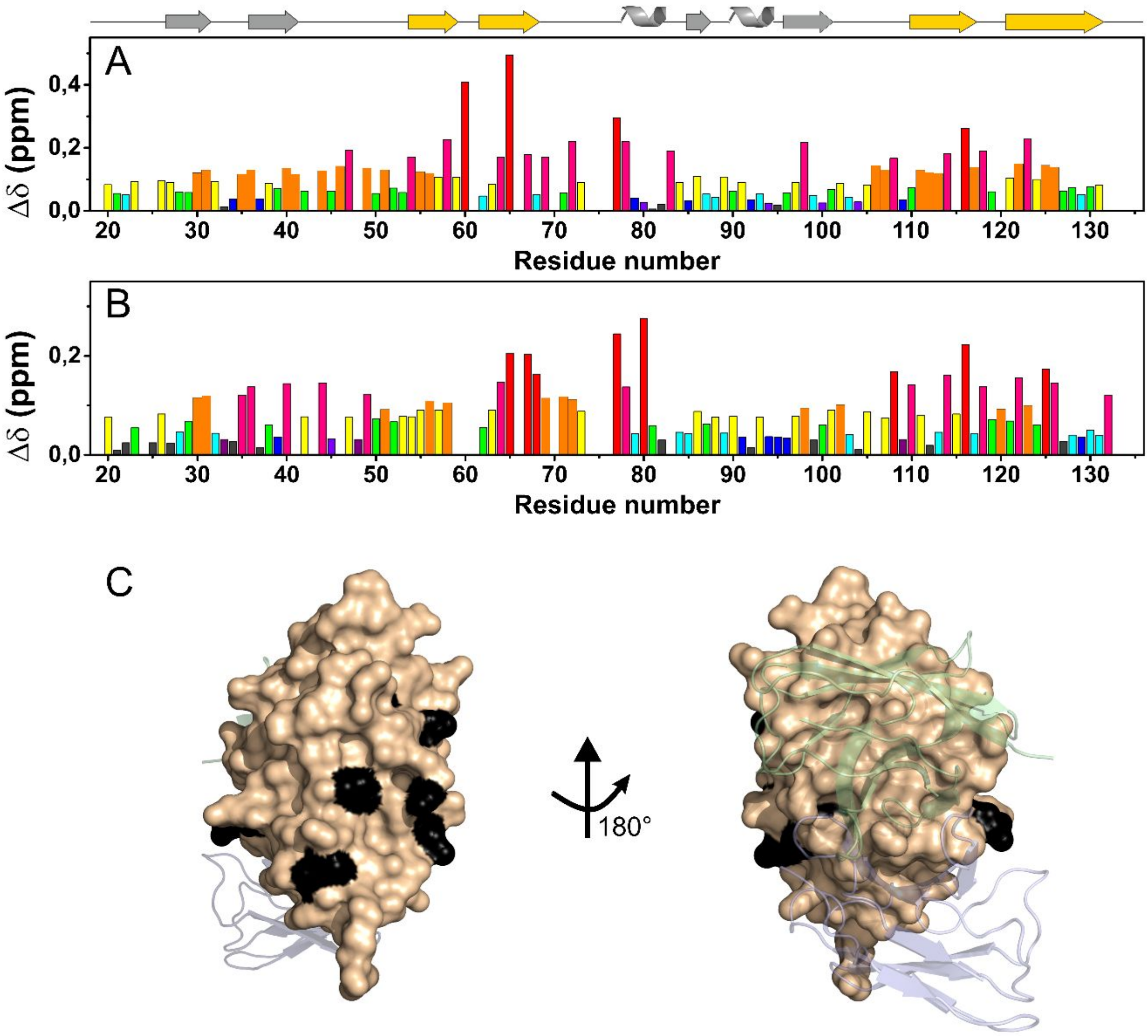

Figure S8. Analysis of CSP with the approach proposed by Schumann and coworkers.[1] The iterative procedure suggested by Schumann and coworkers allowed to refine the threshold for considering interacting and non-interacting residues on CSP evaluated on $^{13}C\alpha$ /^{15}N (A) and ^{1}H /^{15}N (B) resonances values, respectively. The values of CSP larger than three times the standard deviation, defined using the values of CSP of all residues, were removed, and the residual values of CSP used to recalculate a σ_0^{corr} in iterative way, until no CSP value larger than three times of the actual σ_0^{corr} remained. The values removed at each cycle are red, magenta, orange, yellow, green, cyan, blue, violet, purple, respectively. With the new threshold, defined in such a way, most of the CSP are selected as significant, except very few values below this threshold (G33, Y81, R82, G95, according to $^{13}C\alpha$ /^{15}N CSP values, and V21, T22, K25, L27, S34, T37, R82, L92, L99, V104, Y112, T127 according to ^{1}H /^{15}N CSP values). Collectively, the analysis shows that the residues not experiencing a significant CSP are located on regions not interacting with the anti-PD-L1 fusion protein (in black in panel C). The residues corresponding to red, magenta and (most of the) orange selections were identified as significantly shifted also with the threshold considered in figure 7 (>mean + σ).

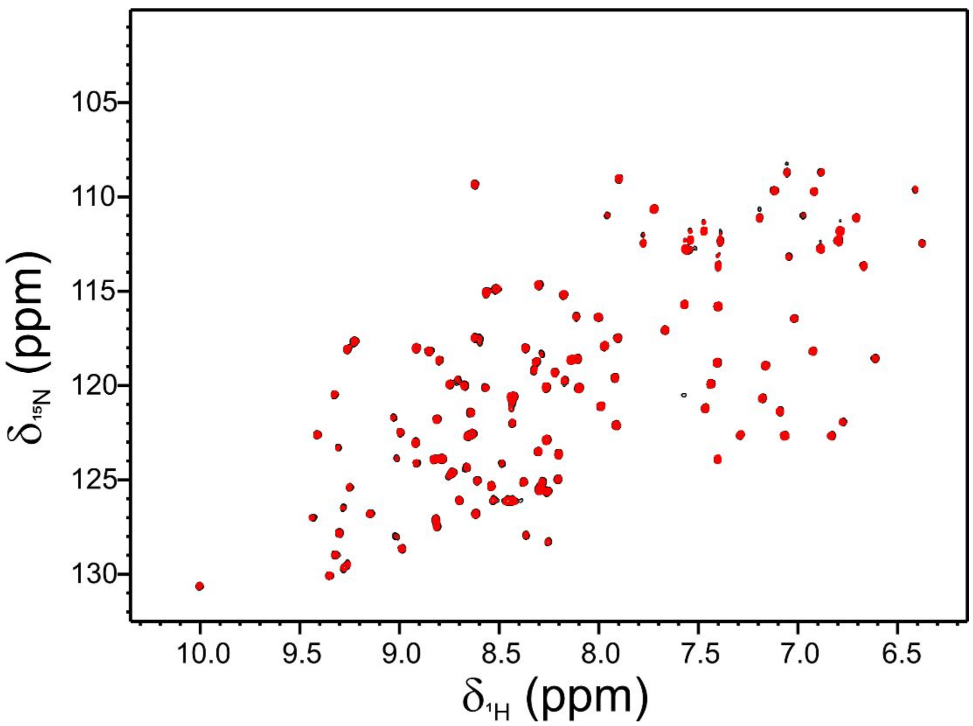

Figure S9. 2D ^{1}H-^{15}N so-FAST HMQC NMR spectra of PD-L1 in the presence of the non-binding mAb (red, 1:0.5, PD-L1: nb-mAb) with respect to free PD-L1 (black). Spectra were acquired at 298 K on a spectrometer operating at 950 MHz (^{1}H Larmor frequency).

Table S1. Parameters used for the acquisition of ^{13}C-detected SSNMR spectra. The number of scans was set according to the amount of material [T= anti-PD-L1 fusion protein; A= non-binding protein; f= free protein].

Experiment	2D NCA	2D NCO	2D DARR
**TD points**<br>**Number of scans**<br>**D1 (sec)**	2048 (f2) x 80 (f1)<br>4096(T)/2048(A)/416(f)<br>1.5	2048 (f2) x 80 (f1)<br>4096(T)/2048(A)<br>1.5	2048 (f2) x 640 (f1)<br>648(T)/480(A)/224(f)<br>1.5
**Transfer 1**<br>**field [kHz]**<br>**shape if applicable**<br>**time [ms]**	HN CP<br>74(H)46(N)<br>100-70 ramp on H<br>1	HN CP<br>74(H)46(N)<br>100-70 ramp on H<br>1	HC CP<br>70(H)42(C)<br>100-70 ramp on H<br>1
**Transfer 2**<br>**field [kHz]**<br>**shape if applicable**<br>**time [ms]**	NCA DCP<br>80(H)35(N)21(C)<br>tangent on C<br>3	NCO DCP<br>80(H)21(N)35(C)<br>tangent on C<br>2	mixing<br><br><br>50

Table S2. Parameters used for the acquisition of ^{1}H-detected SSNMR spectra. The (H)(CA)CB(CA)NH experiment used two spin echoes for the out-and-back CA-CB transfer, with Q3 refocusing pulse selective for the entire ^{13}C aliphatic region of length 150 µs, rf-field peak amplitude 25 kHz, and half-echo delays of 7.2 ms.

Experiment	2D (H)NH	3D (H)CONH	3D (H)CANH	2D ^{1}H-^{13}C plane (H)(CA)CB(CA)NH
**TD points**<br>**Number of scans**<br>**D1 (sec)**	1536 (f2) x 128 (f1)<br>768<br>1.5	1024 (f3) x 42 (f2) x 54 (f1)<br>128<br>1.5	1024 (f3) x 32 (f2) x 64 (f1)<br>256<br>1.5	1024 (f3) x 96 (f1)<br>2896<br>1.5
**Transfer 1**<br>**field [kHz]**<br>**shape if applicable**<br>**time [ms]**	HN CP<br>50(H)10(N)<br>70-100 ramp on H<br>1.6	HCO CP<br>50(H)10(C)<br>70-100 ramp on H<br>4	HCA CP<br>50(H)10(C)<br>70-100 ramp on H<br>2	HCA CP<br>50(H)10(C)<br>70-100 ramp on H<br>2
**Transfer 2**<br>**field [kHz]**<br>**shape if applicable**<br>**time [ms]**	NH CP<br>50(H)10(N)<br>100-70 ramp on H<br>0.6	CON CP<br>35(C)25(N)<br>tangent on N<br>8	CAN CP<br>35(C)25(N)<br>tangent on N<br>5	CACB out and back
**Transfer 3**<br>**field [kHz]**<br>**shape if applicable**<br>**time [ms]**		NH CP<br>50(H)10(N)<br>100-70 ramp on H<br>0.6	NH CP<br>50(H)10(N)<br>100-70 ramp on H<br>0.6	CAN CP<br>35(C)25(N)<br>tangent on N<br>5
**Transfer 4**<br>**field [kHz]**<br>**shape if applicable**<br>**time [ms]**				NH CP<br>50(H)10(N)<br>100-70 ramp on H<br>0.6

Table S3. Assigned $^{13}C\alpha$ and ^{15}N resonances of free PD-L1 from solution NMR and PD-L1 in complex with IgG1 fusion protein by solid state NMR spectra.

		**Free PD-L1 by solution NMR**		**PD-L1 in complex with IgG1 fusion protein by SSNMR**	
**Res type**	**Res num**	**Cα**	**N**	**Cα**	**N**
T	20	60.33	121.87	60.35	122.70
V	21	60.47	127.16	60.27	126.90
T	22	59.85	118.26	59.65	118.19
V	23	56.40	115.17	56.10	115.72
P	24	63.86			
K	25	55.37	113.30		
D	26	53.70	120.54	53.42	121.17
L	27	54.37	122.74	54.01	122.55
Y	28	56.86	126.87	56.69	126.46
V	29	61.81	123.58	61.67	124.05
V	30	59.25	123.97	59.00	122.93
E	31	55.13	120.69	54.91	121.86
Y	32	60.07	125.19	59.71	124.97
G	33	44.96	118.16	44.97	118.04
S	34	57.91	115.78	57.80	116.04
N	35	51.44	115.23	51.19	116.23
M	36	54.32	121.87	54.22	123.15
T	37	61.13	120.20	60.98	120.12
I	38	59.57	122.71	59.34	122.07
E	39	56.21	118.65	56.43	118.20
C	40	55.33	120.20	55.25	118.87
K	41	54.87	123.32	54.54	122.51
F	42	53.67	118.71	53.58	118.13
P	43	62.85			
V	44	61.41	121.16	61.60	122.33
E	45	56.12	125.59	55.92	125.22
K	46	57.90	119.37	57.97	120.78
Q	47	55.58	118.80	54.84	118.35
L	48	55.43	126.22		
D	49	52.20	126.87	51.97	125.64
L	50	57.75	125.35	57.91	125.71
A	51	54.06	117.97	53.72	116.98
A	52	51.35	118.83	51.19	118.23
L	53	55.24	120.81	55.44	121.10
I	54	60.99	128.64	60.39	127.85
V	55	60.85	124.10	60.50	124.96
Y	56	54.70	125.45	54.64	124.27
W	57	56.13	119.78	56.48	119.14
E	58	54.38	121.74	53.66	120.39
M	59	56.24	121.16	56.58	121.80
E	60	58.08	122.50	56.46	122.02
D	61				

K	62	55.51	120.62	55.34	120.83
N	63	55.13	122.55	55.47	122.69
I	64	63.31	124.23	62.88	122.90
I	65	58.56	112.84	56.70	114.56
Q	66	55.76	126.03		
F	67	57.14	129.13	56.70	130.53
V	68	60.88	125.05	60.73	124.69
H	69	56.16	123.78	55.59	122.88
G	70	45.65	102.62		
E	71	54.27	118.29	54.39	117.80
E	72	56.70	123.00	55.82	123.10
D	73	52.99	124.52	52.63	124.70
L	74	55.42	125.10		
K	75	59.49	119.29		
V	76	60.62	109.72		
Q	77	56.46	122.77	56.37	119.84
H	78	59.96	129.64	60.64	128.25
S	79	61.85	122.94	61.72	123.17
S	80	61.07	120.08	61.15	120.27
Y	81	59.62	118.65	59.62	118.59
R	82	58.21	121.45	58.29	121.38
Q	83	58.29	117.67	58.15	115.82
R	84	56.54	116.46	56.24	115.98
A	85	49.99	119.97	49.90	119.75
R	86	54.77	119.97	54.40	120.57
L	87	52.87	123.97	52.75	124.41
L	88	53.94	126.13	53.94	126.56
K	89	59.92	128.10	59.51	127.78
D	90	56.27	118.07	56.25	118.69
Q	91	56.38	115.87	56.06	115.43
L	92	58.31	121.29	58.22	121.04
S	93	61.10	110.68	60.91	110.96
L	94	53.75	119.03	53.67	119.17
G	95	45.34	109.10	45.42	109.12
N	96	51.34	116.52	51.13	116.76
A	97	49.92	130.12	49.62	130.65
A	98	50.75	128.05	51.55	127.19
L	99	52.51	125.71	52.36	125.42
Q	100	53.36	127.16	53.46	127.23
I	101	59.50	127.58	59.38	128.18
T	102	60.37	118.81	60.04	118.56
D	103	54.27	119.65	54.10	119.54
V	104	64.15	118.07	64.03	118.07
K	105	53.40	126.51	53.09	126.72
L	106	59.11	120.10	59.08	121.54
Q	107	57.83	114.63	57.33	114.98
D	108	54.75	117.10	54.49	118.63
A	109	52.59	122.68	52.52	122.38

G	110	44.85	109.49	44.64	110.01
V	111	62.59	121.48	63.08	121.89
Y	112	57.00	130.74	56.57	130.18
R	113	54.59	120.61	55.04	120.19
C	114	51.98	122.06	52.35	123.64
M	115	54.70	124.90		
I	116	59.59	125.03	59.12	122.70
S	117	55.19	120.10	54.64	120.15
Y	118	57.62	128.29	56.98	129.30
G	119	46.49	114.98	46.63	114.49
G	120	43.40	109.75		
A	121	51.42	119.74	51.11	119.04
D	122	53.61	117.52	53.52	116.05
Y	123	55.48	116.42	54.65	115.46
K	124	53.28	118.42	53.60	117.80
R	125	54.88	117.49	54.95	118.93
I	126	60.87	122.68	60.66	121.38
T	127	63.79	124.68	63.65	124.15
V	128	61.39	127.90	61.16	127.45
K	129	55.03	129.76	55.21	129.51
V	130	60.17	122.16	59.94	121.66
N	131	52.10	126.13	51.82	125.73
A	132	50.32	123.71		
P	133	63.07			
Y	134	58.75	124.00		

Table S4. Assigned ^{1}H and ^{15}N resonances of free PD-L1 from solution NMR and PD-L1 in complex with IgG1 fusion protein by solid state NMR spectra. The chemical shifts of both preparations were obtained from the analysis of spectra acquired on [U- ^{2}H, ^{13}C, ^{15}N] isotopically enriched PD-L1.

		**Free PD-L1 by solution NMR**		**PD-L1 in complex with IgG1 fusion protein by SSNMR**	
**Res type**	**Res num**	**H**	**N**	**H**	**N**
T	20	6.70	121.95	6.73	122.69
V	21	9.35	126.98	9.34	126.97
T	22	8.76	118.10	8.72	118.19
V	23	8.47	115.28	8.43	115.77
K	25	6.96	113.16	6.94	113.37
D	26	8.33	120.35	8.36	121.17
L	27	6.98	122.70	7.01	122.53
Y	28	9.07	126.66	8.98	126.63
V	29	8.21	123.48	8.28	124.05
V	30	8.71	123.94	8.60	122.93
E	31	8.34	120.53	8.25	121.64
Y	32	8.28	125.03	8.20	124.85
G	33	9.19	118.01	9.25	117.91

S	34	7.49	115.75	7.50	116.02
N	35	8.09	115.15	7.98	116.23
M	36	8.72	121.77	8.72	123.15
T	37	8.01	120.03	7.98	120.12
I	38	8.57	122.75	8.53	122.17
E	39	6.54	118.51	6.57	118.20
C	40	8.47	120.03	8.64	118.87
K	41	9.22	123.34		
F	42	8.70	118.53	8.57	118.13
V	44	7.88	121.11	7.73	122.33
E	45	8.20	125.54	8.21	125.22
K	46	8.13	119.37		
Q	47	8.04	118.56	8.18	118.33
L	48	8.34	126.04	8.37	126.31
D	49	8.54	126.80	8.62	125.64
L	50	8.45	125.28	8.34	125.74
A	51	7.87	117.79	7.78	116.98
A	52	7.32	118.73	7.41	118.23
L	53	7.10	120.75	7.23	121.13
I	54	8.91	128.62	8.91	127.85
V	55	8.84	124.07	8.87	124.96
Y	56	9.15	125.19	9.27	124.27
W	57	8.63	119.56	8.79	119.14
E	58	8.94	121.57	9.05	120.65
M	59	8.35	121.21		
E	60	8.54	122.64		
K	62	8.35	120.74	8.24	120.83
N	63	8.90	122.34	9.07	122.69
I	64	8.39	124.11	8.56	122.90
I	65	7.46	112.97	7.71	114.59
Q	66	8.50	126.24		
F	67	9.22	128.94	8.97	130.53
V	68	8.19	125.17	7.87	124.74
H	69	8.92	123.67	9.08	122.88
G	70	7.80	102.44		
E	71	6.82	118.26	6.61	117.80
E	72	8.82	123.06	9.04	123.10
D	73	8.55	124.28	8.71	124.70
L	74	8.51	125.00		
K	75	8.23	119.12		
V	76	7.00	109.83		
Q	77	6.73	122.61	6.65	120.20
H	78	9.18	129.33	8.96	128.50
S	79	8.16	122.74	8.20	123.13
S	80	9.47	120.10	10.02	120.27
Y	81	8.02	118.64	8.14	118.59
R	82	7.00	121.32	7.06	121.32
Q	83	9.12	117.63		

R	84	7.92	116.43	7.91	115.98
A	85	7.34	119.88	7.30	120.24
R	86	8.66	119.85	8.76	120.57
L	87	8.73	123.79	8.71	124.41
L	88	8.37	126.01	8.44	126.68
K	89	8.27	127.84	8.19	127.75
D	90	8.81	117.92	8.80	118.69
Q	91	7.30	115.79	7.29	115.43
L	92	7.37	121.17	7.36	121.03
S	93	7.62	110.54	7.75	110.91
L	94	7.07	118.93	7.10	119.26
G	95	7.79	108.99	7.86	109.05
N	96	6.93	116.55	6.88	116.77
A	97	9.27	129.98	9.34	130.66
A	98	8.91	127.81	9.06	127.22
L	99	8.16	125.54	8.10	125.43
Q	100	8.73	126.96	8.74	127.57
I	101	8.72	127.37	8.82	128.12
T	102	8.23	118.69	8.44	118.63
D	103	7.82	119.58	7.74	119.41
V	104	8.27	117.97	8.26	118.07
K	105	9.19	126.32	9.03	126.72
L	106	8.16	119.98		
Q	107	8.20	114.56	8.07	114.94
D	108	7.58	117.02	7.49	118.63
A	109	7.20	122.52	7.15	122.38
G	110	8.53	109.21	8.64	110.50
V	111	8.55	121.50	8.43	122.05
Y	112	9.91	130.36	9.89	130.19
R	113	9.24	120.44	9.32	120.19
C	114	8.35	122.03	8.37	123.64
M	115	8.67	124.80	8.70	123.99
I	116	8.12	124.84	8.24	122.70
S	117	8.59	119.90	8.66	120.16
Y	118	8.16	128.22	8.16	129.60
G	119	8.41	114.83	8.37	114.15
G	120	6.81	109.74	6.73	110.59
A	121	8.07	119.66	8.01	119.04
D	122	8.50	117.57	8.56	116.05
Y	123	8.00	116.33	7.89	115.49
K	124	8.21	118.21	8.30	117.80
R	125	7.81	117.44	7.99	118.93
I	126	9.33	122.60	9.18	121.35
T	127	8.65	124.46	8.67	124.22
V	128	9.22	127.75	9.14	127.86
K	129	9.19	129.53	9.26	129.39
V	130	7.81	122.06	7.75	121.67
N	131	8.61	125.85	8.68	125.73

A	132	8.11	123.50	8.26	124.46
Y	134	7.31	123.91	7.45	124.00

References:

(1) Schumann, F. H.; Riepl, H.; Maurer, T.; Gronwald, W.; Neidig, K.-P.; Kalbitzer, H. R. Combined Chemical Shift Changes and Amino Acid Specific Chemical Shift Mapping of Protein–Protein Interactions. *J Biomol NMR* **2007**, *39* (4), 275–289. https://doi.org/10.1007/s10858-007-9197-z.
